# Complex interplay between neutral and adaptive evolution shaped differential genomic background and disease susceptibility along the Italian peninsula

**DOI:** 10.1038/srep32513

**Published:** 2016-09-01

**Authors:** Marco Sazzini, Guido Alberto Gnecchi Ruscone, Cristina Giuliani, Stefania Sarno, Andrea Quagliariello, Sara De Fanti, Alessio Boattini, Davide Gentilini, Giovanni Fiorito, Mariagrazia Catanoso, Luigi Boiardi, Stefania Croci, Pierluigi Macchioni, Vilma Mantovani, Anna Maria Di Blasio, Giuseppe Matullo, Carlo Salvarani, Claudio Franceschi, Davide Pettener, Paolo Garagnani, Donata Luiselli

**Affiliations:** 1 Laboratory of Molecular Anthropology and Centre for Genome Biology, Department of Biological, Geological and Environmental Sciences, University of Bologna, Bologna, Italy; 2 Centre for Biomedical Research and Technologies, Italian Auxologic Institute, IRCCS, Milano, Italy; 3 Department of Medical Sciences, University of Torino and Human Genetics Foundation, Torino, Italy; 4 Rheumatology Unit, Arcispedale Santa Maria Nuova Hospital, IRCCS, Reggio Emilia, Italy; 5 Allergy and Advanced Biotechnologies Unit, Arcispedale Santa Maria Nuova Hospital, IRCCS, Reggio Emilia, Italy; 6 Center for Applied Biomedical Research, S. Orsola-Malpighi University Hospital, Bologna, Italy; 7 Department of Experimental, Diagnostic and Specialty Medicine, University of Bologna, Bologna, Italy

## Abstract

The Italian peninsula has long represented a natural hub for human migrations across the Mediterranean area, being involved in several prehistoric and historical population movements. Coupled with a patchy environmental landscape entailing different ecological/cultural selective pressures, this might have produced peculiar patterns of population structure and local adaptations responsible for heterogeneous genomic background of present-day Italians. To disentangle this complex scenario, genome-wide data from 780 Italian individuals were generated and set into the context of European/Mediterranean genomic diversity by comparison with genotypes from 50 populations. To maximize possibility of pinpointing functional genomic regions that have played adaptive roles during Italian natural history, our survey included also ~250,000 exomic markers and ~20,000 coding/regulatory variants with well-established clinical relevance. This enabled fine-grained dissection of Italian population structure through the identification of clusters of genetically homogeneous provinces and of genomic regions underlying their local adaptations. Description of such patterns disclosed crucial implications for understanding differential susceptibility to some inflammatory/autoimmune disorders, coronary artery disease and type 2 diabetes of diverse Italian subpopulations, suggesting the evolutionary causes that made some of them particularly exposed to the metabolic and immune challenges imposed by dietary and lifestyle shifts that involved western societies in the last centuries.

The Italian peninsula represents a fascinating case study for biological anthropologists due to the role played as natural hub for human migrations across the Mediterranean area during both prehistoric and historical periods.

Since early Upper Paleolithic spread of anatomically modern humans from the Levant into the European continent, the southern heel of the peninsula was reached by coastline migrations (~45 kya) that predated population movements associated to Aurignacian culture diffusion through the Balkans and Central Europe^1^. Also during Last Glacial Maximum (LGM, 25–19 kya), when ice sheets or permafrost covered most of Northern/Central Europe, Central and Southern Italy plausibly represented one of the few refuge areas where human groups contracted and from which re-peopled the whole continent^2^,^3^,^4^,^5^,^6^. The peninsula then appeared to be involved in migratory events that enabled diffusion of agricultural techniques from the Aegean Sea to Western Europe^7^. Southern coastline routes followed by people belonging to Impressa and Cardial cultures brought the Neolithic in Southern Italy considerably earlier (~6 kya) than in other European regions^8^,^9^. The northern and western parts of the peninsula were instead reached only some centuries later by demic flow associated to the Linearbandkeramik culture and originated along the Vardar-Danube-Rhine corridor^3^,^10^. Furthermore, a series of demographic and social events occurred during Metal Ages are supposed to have led to the origin of several proto-historic Italic populations^3^,^11^. In addition to these prehistoric demographic processes, a cascade of historical events could have determined appreciable gene flow to and within Italy. In particular, such migrations trace their origins back to the establishment of Phoenician and Greek colonies in Western Mediterranean (~2.8 kya), to the Roman Empire expansion (2.3–1.8 kya) and the Arab occupation of Sicily (1.3 kya) (see Sazzini *et al.*^12^ for a review), as well as to more recent events^13^,^14^. Accordingly, both paleoanthropological, archaeological/historical and genetics records agree in pointing to the Italian peninsula as a territory involved in large- and small-scale population movements stratified along a wide temporal interval and that likely reshuffled local patterns of genomic variation multiple times.

In addition to this complex demography, also a patchy environmental landscape characterized by different bioclimates^15^ might have contributed in shaping a heterogeneous genomic background for the overall population distributed along the peninsula. In fact, it could have entailed a mosaic of selective pressures plausibly related to differential nutritional resources and pathogens distribution able to trigger local adaptive events.

To date, several studies based on the analysis of classical and uniparentally-inherited markers provided evidence of appreciable Italian population structure, especially as concerns Y-chromosome lineages, while a more homogeneous mitochondrial DNA background was described^3^,^16^,^17^,^18^,^19^. Nevertheless, few autosomal genome-wide datasets have been generated until now to further disentangle this scenario and to explicitly test hypotheses related to occurrence of local adaptive events in diverse Italian subpopulations^20^,^21^,^22^. In fact, genome-wide data suggested the existence of potential genetic barriers between Italians and other Europeans^23^ and further corroborated evidence for internal differentiation of the overall Italian population^14^,^22^,^24^. However, most of these surveys were focused on single subpopulations (mainly Sardinians) or were constrained by limited sample sizes and/or inclusion of subjects collected within the framework of European GWASs, thus not chosen according to stringent bio-anthropological criteria.

To overcome these issues, we generated data for a panel of samples that enables to describe the Italian population with an unprecedented level of geographical resolution. For this purpose, 780 individuals were selected to be representative of variation observable at 20 provinces equally distributed in four geographical macro-areas (i.e. Northern Italy, N_ITA; Central Italy, C_ITA; Southern Italy, S_ITA, and Sardinia, SARD) for which previous studies suggested relatively high internal historical/cultural homogeneity^3^,^25^. In addition to ~280,000 genome-wide SNPs, our survey included also ~250,000 exomic markers and ~20,000 variants at coding/regulatory chromosomal intervals with well-established clinical relevance. This maximized chances to pinpoint functional genomic regions with potential adaptive roles, as well as to investigate a realistic approximation of the full spectrum of allele frequency due to genotyping of relatively rare variants. This approach enabled to fine dissect population structure observable across a so circumscribed geographical area and to infer local adaptations, some of which are potentially responsible for differential disease susceptibility of diverse Italian subpopulations.

## Results and Discussion

### Fine Dissection of the Italian Population Structure

**Figure 1:**
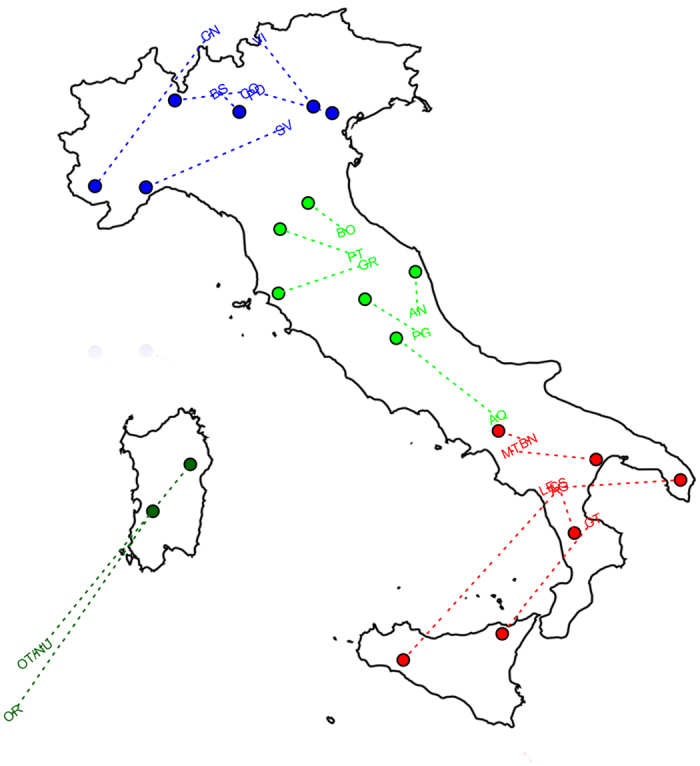
Procrustes analysis projecting genomic information summarized by first and second PCs (population codes) onto geographic coordinates of the sampled Italian provinces (circles). Colors indicate population clusters suggested by geographic coordinates, while dotted lines visualize residuals of genetic/geographic regression. Provinces clustering within the N_ITA, C_ITA, S_ITA and SARD groups are displayed in blue, green, red, and dark green, respectively. Procrustes analysis and map of the Italian peninsula were plotted using the R software v.3.1.1 (R: A Language and Environment for Statistical Computing, R Core Team, R Foundation for Statistical Computing, Vienna, Austria (2016) https://www.R-project.org).

After quality control (QC) procedures, 524,738 autosomal markers were retained for 747 individuals from 20 provinces belonging to N_ITA, C_ITA, S_ITA, SARD and distributed as described in Fig. 1 and Supplementary Table S1. Descriptive analyses aimed at summarizing patterns of population structure were performed on 135,905 SNPs selected by excluding variants in LD. A filtered panel of 737 individuals resulting from exclusion of outliers with respect to the bulk of subjects collected at their province of origin (Supplementary Information) was used for these and subsequent analyses.

Principal Components Analysis (PCA) highlighted clear differentiation of peninsular Italians from Sardinians according to PC1 (0.25% of variance) and distribution of peninsular variation along PC2 (0.22%) in line with the north-south cline already described for Y-chromosome lineages^3^,^18^ and autosomal loci^3^,^22^,^24^ (Supplementary Fig. S1). Although populations from examined provinces displayed appreciable internal variability, they largely overlapped only with other groups belonging to the same macro-geographical area, with intersection between samples from diverse macro-areas being limited to few sampling locations (Supplementary Information), especially to L’Aquila (C_ITA), most of whose subjects clustered within the bulk of southern samples.

**Figure 2:**
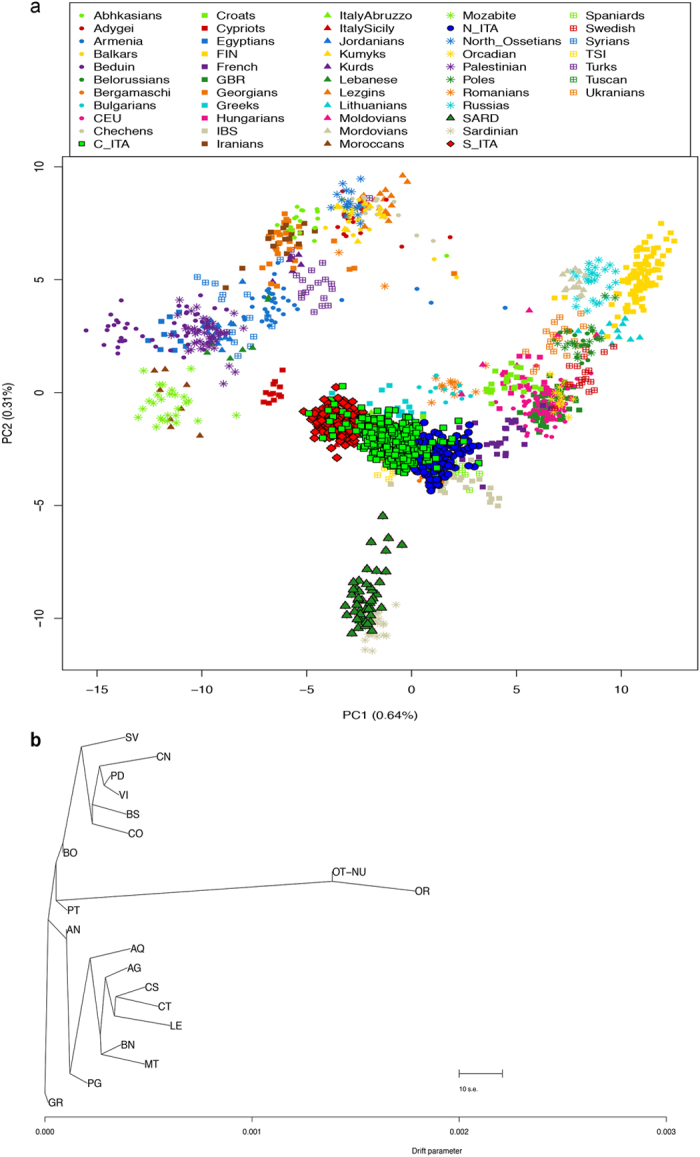
Principal components and TreeMix analyses performed on the Italian and extended datasets. (**a**) First and second PCs computed on the extended dataset including the four Italian population clusters and 47 European and Mediterranean human groups. Samples clustering within the N_ITA, C_ITA, S_ITA and SARD groups are displayed in blue, green, red, and dark green, respectively. Abbreviations for the reference populations as reported in Supplementary Table S1. (**b**) TreeMix graph describing the splitting pattern without migration observed in the Italian dataset. The length of the branches is proportional to the genetic drift experienced by each population.

This pattern was confirmed when PCA was extended to 1,282 additional individuals from 50 European/Mediterranean populations (Supplementary Table S1) by considering 55,029 LD-pruned SNPs, showing a distribution of genetically related populations that roughly reflected the geographic map of Europe (Fig. 2a). PC1 (0.64%) enabled to differentiate populations along a north-south cline in accordance to previous reports^23^,^26^, while PC2 (0.30%) described a longitudinal axis of variation. This picture also matches the southeast-northwest gradient of European diversity depicted by classical and Y-chromosome markers^17^,^27^,^28^ and confirmed that even within a broad geographical context a latitudinal cline of variation was observable along the Italian peninsula (Supplementary Information).

Despite such a diversity gradient, relatively well-defined population clusters could be distinguished along the peninsula and their reliability was tested by projecting genomic information summarized by the two most informative PCs onto geographic coordinates via Procrustes analysis (Fig. 1). Although overall similarity between genomic and geographic structures was found (t_0_ = 0.81, p = 9.9 × 10^−6^), mismatches between computed eigenvectors and longitude/latitude information were pointed out by analysis of residuals (Supplementary Information), mainly indicating that the considered provinces tend to be closer genetically than geographically within both N_ITA and S_ITA macro-areas. Populations from C_ITA instead turned out to be more heterogeneous, especially due to the sample from L’Aquila that showed higher affinity to southern populations than to other central ones (Fig. 1). High homogeneity within each macro-area, especially S_ITA and SARD, was confirmed also by Discriminant Analysis of Principal Components (DAPC) applied to population clusters suggested by PCA and Procrustes analysis (i.e. by considering L’Aquila as part of the S_ITA cluster) (Supplementary Fig. S2 and Supplementary Information).

Construction of a TreeMix maximum likelihood tree provided independent inference of splitting events occurred among Italian subpopulations, describing a pattern without migration that roughly reflected the latitudinal cline pointed out by previous analyses (Fig. 2b and Supplementary Fig. S3). As expected, SARD showed the highest level of drift from the ancestral population and branched out from the part of tree from which also most C_ITA provinces split. Relationships among these C_ITA samples were considerably less tight than those observable within N_ITA and S_ITA clusters. Provinces from N_ITA and S_ITA indeed grouped into two well-defined clades clearly reflecting their geographical distribution, with the latter including also people from L’Aquila, in accordance to results from Procrustes and DAPC analyses (Supplementary Information).

### Inferring Admixture Events between Italian and European/Mediterranean populations

TreeMix was also used to construct trees by allowing for increasing migration edges (m), highlighting a series of potential admixture events between Italian subpopulations, as described in Supplementary Fig. S4 and Supplementary Information.

The same approach was then applied to the extended dataset to infer potential gene flow between clusters of Italian provinces and several European/Mediterranean populations, pointing to six admixture events that minimized population pairs with poor fits of the model to the data. Accordingly, appreciable gene flow was observed from different Middle Eastern and North African populations to SARD, C_ITA and S_ITA, with especially the latter group still showing the most detectable signatures of migration (Supplementary Fig. S5 and Supplementary Information). These findings were in agreement with results obtained by formally testing for admixture with *f3* and ALDER algorithms, with the most significant Z-scores pointing to S_ITA and C_ITA as results of admixture between Western/Eastern European and Northern African or Middle Eastern populations. The most relevant score related to N_ITA instead suggested admixture between S_ITA and Eastern Europeans (Supplementary Information). ALDER also provided rough time estimates for inferred admixture events, dating gene flow from North Africa to S_ITA at 0.7–0.5 kya, that from the Middle East to C_ITA at 1.5–0.7 kya, as well as that from Eastern Europe to N_ITA at ~2 kya (Supplementary Table S2 and Supplementary Information). This picture suggests that the overall Italian population is characterized by a heterogeneous network of genomic relationships involving human groups from both the north-western and south-eastern far ends of the Mediterranean basin. In particular, people from N_ITA appeared to be more closely related to Western and Eastern European populations, while moving southwards along the peninsula a progressive genetic connection with Middle Eastern and Northern African populations emerged.

**Figure 3:**
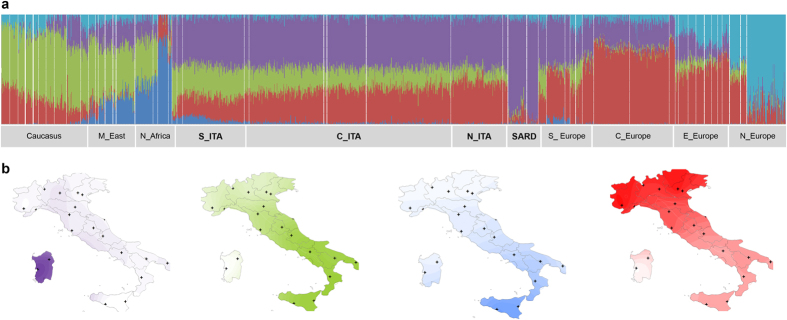
ADMIXTURE analysis performed on the extended dataset. (**a**) Ancestry proportions for each individual (columns) at K = 5 estimated by hypothesizing five hypothetical ancestral populations, that is the number of groups enabling the best predictive accuracy of the model according to cross-validation procedure. Stacked barplots were generated using the R software v.3.1.1 (https://www.r-project.org). (**b**) Interpolation maps showing spatial distribution along the Italian peninsula of the four most represented ancestry proportions observed in the Italian genomes at K = 5. Kriging interpolation and maps of the Italian peninsula were plotted using Surfer^®^ [7] from Golden Software, LLC (www.goldensoftware.com).

To evaluate at a finer geographical scale the contribution of inferred admixture events to the overall Italian variation, ancestry proportions for each individual were estimated by testing the existence of two to ten hypothetical ancestral populations via ADMIXTURE. The best predictive accuracy was achieved by the model when four/five ancestral groups (K = 4 or K = 5) were hypothesized (Supplementary Figs S6 and S7). Five ancestry components were thus identified in the whole dataset and interpolation maps were used to show spatial distribution of the four most represented along the Italian peninsula (Fig. 3a,b). The purple component was predominant in Southern European groups and equally distributed along the peninsula (average frequency of 46%), almost reaching fixation in Sardinians (85%) plausibly due to their long-term isolation especially to Post-Neolithic processes^29^. This further corroborates the hypothesis of an ancestry fraction proper of Sardinians that potentially represents a relic signature of the very early Neolithic European genomic background, as previously discussed for TreeMix/*f3* results (Supplementary Information) and as suggested by data on ancient Mediterranean genomes ascribable to the Cardial culture^30^. The green component was considerably represented in samples from Caucasus and Middle East, being also evident in some Southern European populations (e.g. Greeks) and, especially, in Southern Italy (28%), progressively decreasing towards the northern part of the peninsula (12%). A similar, but even more extreme south-north gradient was observed also for the blue component highly representative of Northern African groups that was additionally detected in Middle East and, to a significant lower extent, in Southern Italy (4.6%, mainly in Sicily). Distributions of these two ancestry fractions suggest that they plausibly reflect the combined impact of multiple population movements stratified along a wide time interval, but broadly following coastline migratory routes towards Southern Italy. The Middle Eastern-like ancestry could be the complex result of events spanning from earlier arrival of Neolithic farmers in Southern Italy than in Northern Italy^7^,^8^, in line with emerging genetic evidence supporting early Neolithic colonization routes following northern Mediterranean coastlines^10^,^30^,^31^, to more recent historical processes^12^,^14^,^32^,^33^. Results from ALDER analysis actually suggest that gene flow from the Middle East interested C_ITA until more recent times, pointing to an admixture signature plausibly ascribable to events occurred during recurrent expansions and contractions of the Byzantine Empire (1.7–0.5 kya)^28^ (Supplementary Table S2). The same analysis supported also the hypothesis that the Northern African-like ancestry represents a genetic footprint related to the Arab occupation of Sicily (1.3–0.7 kya)^13^,^14^ (Supplementary Table S2). The red component characterized most of Central and Eastern European populations, being reduced in Sardinia (7.4%) and showing a decreasing north-south gradient in peninsular Italy (from 39% in N_ITA to 20% in S_ITA). According to the proposed impact of Late Neolithic and Bronze Age population movements on the genetic landscape of present-day Europeans^34^,^35^, we can speculate that this component is associated, at least in part, to these large-scale demographic processes that connected Central/Eastern and Western Europe since ~4.5 kya. Nevertheless, the absence of a Yamnaya-related genetic signature in the northern Italian Copper Age Remedello specimen suggests that these migrations could have reached the Italian peninsula considerably later^34^. Moreover, roughly overlapping migrations occurred during the expansion of the Roman Empire or in Medieval times could have also reinforced and/or re-shaped this pattern^12^,^13^, with the former events being the most plausible responsible for the signature observed in N_ITA according to time estimates obtained by ALDER analysis (Supplementary Table S2). Finally, the light blue component was observed in eastern and northern European populations, being predominant in Finn plausibly due to high genetic drift, but reached equal and negligible frequencies in Italian groups (2.8%). Evidence for a structured European meta-population since ~36 kya, implying different pre-Neolithic contribution to the northern and southern European genomic background^36^, coupled with recent identification of two distinct European genetic clusters (i.e. the Věstonice and the Villabruna ones) associated respectively with the Gravettian culture and with expansion of people from the Middle East since ~14 kya^6^, could have laid foundations for such a pattern. This could explain low, but detectable percentage of this ancestry component in the overall Italian population, also in agreement with the hypothesis of a reservoir of ancient variation represented by the Italian peninsula during LGM^5^.

### Signatures of Natural Selection on the Italian Genomes

From the described picture of population structure it emerges that broad clusters of genetically homogeneous provinces could be distinguished within the overall Italian population, each one reflecting peculiar demographic history. To gain first hints about other evolutionary processes that contributed to shape this mosaic genomic landscape, functional properties of chromosomal regions driving the observed pattern of differentiation were evaluated to test whether they are randomly distributed or enriched for specific gene functions. Single locus F_st_ was computed on 333,691 polymorphic SNPs for each pairwise cluster comparison and variants scoring in the top 1% of F_st_ distribution in each Minor Allele Frequency (MAF) bin (Supplementary Table S3) were retained as highly differentiated loci and submitted to functional annotation clustering (Supplementary Table S4). The most relevant findings emerged from N_ITA and S_ITA comparison, which showed significantly enriched Gene Ontology (GO) terms among the most differentiated genes being associated to processes of cell/neuron recognition and projection organization, as well as to cellular components devoted to signalling/trafficking (e.g. membrane rafts) that play a role in the development of pathological conditions such as Alzheimer’s, Parkinson’s and cardiovascular diseases^37^ (Supplementary Information).

These results suggest that diverse selective pressures could have triggered local adaptations along the peninsula contributing to differentially shape variation at specific functional pathways. Such a hypothesis was corroborated by testing for different footprints of natural selection on diverse Italian population clusters through the identification of chromosomal regions showing both extreme differentiation along the peninsula and unusually extended haplotype homozigosity (Supplementary Table S5). The most plausible genomic intervals having undergone positive selection in N_ITA encompassed genes or regulatory sequences at which neutral evolution was inferred for remaining population clusters. N_ITA specific signatures emerged from loci implicated in signalling cascades that regulate adipogenic processes in response to fat dietary intake (*WDPCP*) and HDL cholesterol concentration (*RNMTL1P2*), thus contributing to reduced risk for coronary artery disease (CAD) and type 2 diabetes (T2D) (see Supplementary Information for more details and references). Accordingly, we can speculate that selective pressures geographically restricted to N_ITA acted mainly on modulators of lipid metabolism, being plausibly related to temperate climate conditions and consequent adoption of calorie-rich diets. Otherwise, none of the genomic windows identified as potential targets of selection in C_ITA and S_ITA were specific of a single population cluster. The most relevant signatures involved loci associated to small height variation, but playing a role in immune specialization (*DLEU1* lincRNA), reduced risk of myeloma and optimal antibodies production by B-lymphocytes (*TNKS* enhancer) or able to modulate responses to mycobacterial infections (*IL23R*) (see Supplementary Information for more details and references). Coupled with paleoanthropological evidence of long coexistence between populations settled along the Italian peninsula and mycobacteria responsible for tuberculosis and leprosy^38^, this finding suggests that such pathogens might have represented selective pressures able to appreciably shape variation in most Italian groups. Such a signature was particularly robust in S_ITA and drove alleles associated to increased risk of inflammatory bowel and Crohn’s diseases at considerably higher frequency in these populations (45%) than in other Italian ones (30%). Moreover, less outstanding signatures were found to be shared among all Italians but SARD. They involved loci implicated in modulation of inflammatory reactions (*TLR10*-*TLR1*-*TLR6* cluster) or MHC class III alleles that regulate peptides presentation to immune effectors and functionality of lymphocyte antigen complex, but that also increase susceptibility to systemic lupus erythematosus (*BAG6* and *LY6G6C*) (see Supplementary Information for more details and references). Finally, the most plausible candidates having undergone positive selection exclusively in SARD encompassed two main genomic regions. They are known to modulate erythrocytes sedimentation rate and inter-individual variation in the severity of malaria (*CR1)*, but entailing different alleles than those proved to modify host disease susceptibility in African populations^39^, and production of pro-inflammatory and oxidative metabolites involved in the pathogenesis of asthma and malaria (*MS4A2*) (see Supplementary Information for more details and references). Accordingly, selective pressures having acted on Italian populations from Mediterranean regions seem to have targeted mainly immune pathways, being thus plausibly related to peculiar pathogens landscapes. Moreover, some of these past adaptive events led to evolution of efficient responses to such pathogens that however contribute to increased susceptibility to autoimmune or inflammatory disorders in present-day Italians.

### Additional Loci Influencing Differential Disease Susceptibility Along the Italian Peninsula

Variants characterized by extremely unusual differentiation along the peninsula and thus plausibly underlying sharp distinction of adaptive events occurred in diverse Italian regions were further shortlisted by filtering for loci with significant difference in derived allele frequency (∆DAF). Only when N_ITA and S_ITA clusters were contrasted, four SNPs showed significant ∆DAF (p < 3.19 × 10^−8^, Table 1) considerably close to the threshold (0.25) used to pinpoint highly differentiated loci among intra-continental populations^40^ (Supplementary Information). However, when correction for background differentiation between populations was applied, obtained p-values considerably increased (p < 2.53 × 10^−04^), suggesting that demographic processes played an appreciable role in shaping the observed ∆DAFs in addition to putative different selective pressures.

**Figure 4:**
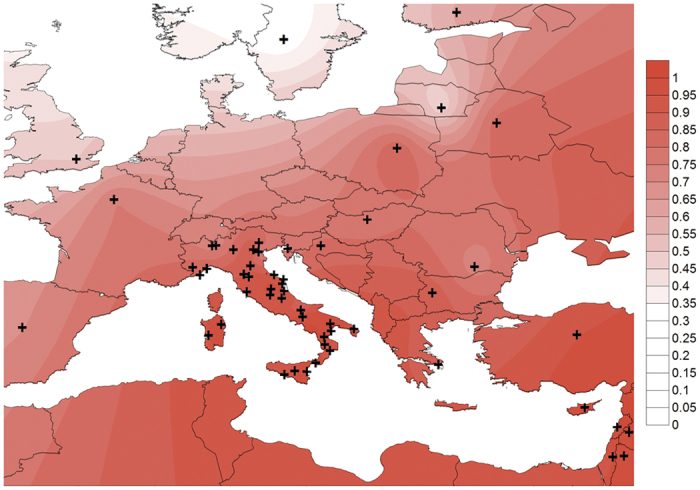
Interpolation map showing frequency gradient of the T2D-associated rs6723108 ancestral allele across a set of Mediterranean and European populations. Kriging interpolation of rs6723108 ancestral allele frequency and geographic coordinates of the studied Italian provinces, as well as of the additional Italian samples used for replication experiments and for a set of reference populations. Kriging interpolation and geographical map were plotted using Surfer^®^ [7] from Golden Software, LLC (www.goldensoftware.com).

Nevertheless, these findings offered a further opportunity to shed lights on some of the medical implications that are deeply rooted in the genetic history of Italians, as well as on their possible evolutionary determinants. This was enabled by disentangling complex interplay between demographic and selective forces having acted on SNPs unusually differentiated between the opposite geographical ends of the peninsula through the reconstruction of their recent evolutionary trajectories. Among them, rs7570971 and rs17261772 map on the *RAB3GAP1* gene (Table 1) involved in presynaptic neurotransmitter release/recycling and contributing to maintenance of neurotransmission, but showed low LD with each other (r^2^ = 0.31). The rs7570971 derived allele is highly represented in western and northern European populations (e.g. CEU, 71%; GBR, 68%; FIN 59%; IBS, 44%) plausibly due to positive selection (CEU iHS = −4.129; whole *RAB3GAP1* p = 2.1 × 10^−04^)^41^. It showed instead lower and southward decreasing occurrence along the Italian peninsula, being significantly over-represented in N_ITA (27%) than in S_ITA (8%) and showing intermediate, but not significantly reduced frequency in C_ITA (14%). Therefore, remarkable frequency of the non-selected ancestral allele was observed in the overall Italian population. This allele is strongly associated to metabolic markers of impaired glycaemic control in T2D and to total cholesterol levels, representing a genetic risk factor also for CAD^42^,^43^. A similar cline was observed also for the rs17261772 derived allele, whose frequency drops from an average of 68% in European populations to 46% in N_ITA, 33% in C_ITA, 31% in SARD and 23% in S_ITA. The other SNPs showing significant ∆DAF were rs1446585 and rs6723108 (Table 1). The former maps on the *R3HDM1* gene involved in interactions with single-stranded DNA/RNA that is supposed to play a role in mRNA stabilization and whose variants are associated to increased triglycerides levels^42^,^44^. Distribution of this SNP reflected the north-south differentiation gradient of rs7570971, plausibly due to their moderated LD (r^2^ = 0.65). In fact, frequency of the rs1446585 derived allele ranges from an average of 65% in Europeans to 34% in N_ITA, 21% in C_ITA, 12% in S_ITA and 7% in SARD. Finally, rs6723108 maps near the *TMEM163* gene encoding for a vesicular transporter expressed in synapses and its ancestral allele is associated to insulin resistance and increased T2D risk^45^. Due to its low LD with rs7570971 (r^2^ = 0.44) it could represent a second independent signal of differentiation between N_ITA and S_ITA entailing medical implications. Frequency of its derived allele drops from an European average of 43% to 20% in N_ITA, 11% in C_ITA, 8% in S_ITA, 3% in SARD (Fig. 4) and was proved to be influenced by positive selection in CEU (iHS = −3.189; whole *TMEM163* p = 4.9 × 10^−03^)^41^. To confirm the described frequency patterns, distribution of rs6723108 and rs1446585 (considered as a tag also for rs7570971) in peninsular Italians was validated by genotyping 380 additional individuals (Supplementary Table S1), pointing to significant N_ITA/S_ITA ∆DAF (20% vs. 7%, p = 1.68 × 10^−2^ and 33% vs. 12%, p = 1.71 × 10^−3^). Ancestral disease-associated alleles at these loci resulted significantly over-represented also in most of the examined Mediterranean and Middle Eastern populations (e.g. Greek, Cyprus, Turkey, Armenia, Georgia, Iran, Syria, Lebanon, Jordan, Palestine) in addition to Southern Italy (Fig. 4). Overall, this south-north decreasing frequency matches that of T2D incidence along the Italian peninsula (i.e. twofold values in S_ITA than in C_ITA/N_ITA, http://www.istat.it/it/archivio/71090). This finding is in line with maintenance of such alleles at high frequency in S_ITA and SARD due to the absence of selective pressures having acted on their complementary derived alleles (iHS = −0.90 and iHS = −0.73). Instead, moderate frequency of derived alleles in N_ITA and C_ITA might be due to weaker, but appreciable, positive selection having acted on them (iHS = −2.54 and iHS = −2.50) with respect to what observed in most western European populations. Since these loci are located on chromosome 2 at 201 kb to 1.13 Mb from the −13,910 *LCT* adaptive variant, we can also hypothesize that their frequency gradient along the Italian peninsula reflects a hitchhiking effect related to the action of positive selection on the *LCT* promoter. Unfortunately, the −13,910 *LCT* SNP was not assayed by the genotyping array used in the present study and this prevents to directly estimate LD values between it and the identified candidate variants in the examined population groups. However, when compared to northern European patterns the high-LD block surrounding the −13,910 SNP is known to be significantly shorter in populations from both Northern and Southern Italy^10^. In the overall Italian population, such loci could thus map beyond the extended *LCT* haplotype targeted by selection. When TSI were considered as a rough approximation of the Italian people, the hitchhiking hypothesis seems to be corroborated for rs7570971, which is in strong LD with the −13,910 *LCT* SNP (r^2^ = 0.94), but not for rs1446585, rs6723108 and rs17261772, which instead show moderate to low LD with it (r^2^ = 0.60, r^2^ = 0.35 and r^2^ = 0.23, respectively). Nevertheless, complete independence of the observed unusual differentiation at the above-mentioned loci from evolution of the lactase persistence phenotype cannot be definitively confirmed. In fact, according to the available data, it is virtually impossible to disentangle between different scenarios entailing hitchhiking effects and/or selection having targeted multiple adaptive alleles at different genes, including also, but not only, the −13,910 *LCT* SNP.

Despite that, over-representation of these ancestral alleles associated with high cholesterol levels and insulin resistance only in S_ITA appears to be in line with results obtained by F_st_/iHS analyses, which suggest potential adaptive evolution of modulators of lipid metabolism in N_ITA. In addition to the concomitant selective force represented by milk consumption, increased frequency of their complementary derived alleles in Northern/Western Europe, and to a lesser extent in N_ITA, might have been prompted also by natural selection in response to other dietary-related pressures. In fact, human groups expanding from the Levant into the European continent were forced to cope with new nutritional resources and with progressively colder environments, being also more exposed to climate fluctuations occurred in Eurasia since the Late Mesolithic and resulting in periodic decades of extremely cold winters^46^. Consequently, these populations were likely subjected to important dietary shifts, for instance towards high-calorie/high-fats diets^47^,^48^. A reduction of the potential side effects of such new dietary regimens, plausibly ensured by these derived alleles, could have thus played a crucial adaptive role. According to this view, putative selective pressures at the described loci likely arose along southeastern-northwestern continental migratory routes on the source human groups from which the majority of present-day western Europeans derived most of their genomic ancestry. Such a scenario could explain the wide distribution of these adaptive alleles across Northern and Western Europe, as well as their reduced occurrence in Italy, especially in southern regions, in accordance to substantial contribution to the Italian genomic variation provided by southern coastline population movements involving human groups that were not subjected to these selective pressures.

## Conclusions

Despite evident cline variation distributed along the Italian peninsula, broad clusters of genetically homogeneous provinces could be distinguished and local adaptive events were proved to have influenced their differentiation in addition to appreciably dissimilar demographic histories. Furthermore, complex interplay between neutral and adaptive evolution appeared to have determined also differential disease susceptibility of Italian subpopulations, making some of them more prone to develop CAD, T2D and some inflammatory/autoimmune diseases, in the context of the novel metabolic and immune challenges imposed by modern lifestyles. Long-term absence or reduction of specific dietary-related selective pressures on most Italian subpopulations with respect to the bulk of western Europeans, as well as prolonged coexistence with mycobacteria responsible for tuberculosis and leprosy, coupled with a more extensive gene flow from Southern Europe and the Middle East, have maintained alleles responsible for increased cholesterol levels, insulin resistance and aggressive inflammatory responses to pathogens at considerable frequency in the Italian gene pool. Therefore, the peculiar evolutionary history of Italian people, which entails elements proper of both continental and Mediterranean Europeans admixed and distributed in a restricted but heterogeneous geographical area, was proved to have made them particularly exposed to the metabolic/immune challenges and resulting health implications related to the adoption of modern diets and sedentary lifestyle or to introduction of completely new dietary immune-stimulatory epitopes^49^ that involved western societies in the last centuries. These findings provided suggestive evolutionary medicine case studies in which genetic susceptibility to certain diseases seems to be mediated by alleles targeted by natural selection in the past, but having become detrimental in present-day populations due to recent and substantial cultural shifts.

## Materials and Methods

### Samples Collection and Genotyping

A total of 780 unrelated healthy individuals from 20 provinces distributed along the Italian peninsula, Sicily and Sardinia, were selected among those recruited during the sampling campaign described in Boattini *et al.*^3^ to maximize the amount of observable genomic variation and by focusing on subjects at least three-generations native of a given province (i.e. with all grandparents originating from the same province). Detailed description of the sampling strategy was provided in Boattini *et al.*^3^, while information about provinces and samples included in the present study are reported on Supplementary Table S1.

Informed consent related to donation of blood specimens to be processed for extraction of de-identified DNA samples to be used for population genomics analyses was obtained from all participants within the framework of the study by Boattini *et al.*^3^ (protocol n. 85/2009/U/Tess approved by the Bologna S.Orsola-Malpighi University Hospital ethics committee). The present study was designed and performed in accordance with relevant guidelines and regulations and according to ethical principles for medical research involving human subjects stated by the WMA Declaration of Helsinki. Further approval for this study was also released in January 2011 by the Azienda-Ospedaliera Arcispedale Santa Maria Nuova ethics committee (Reggio Emilia) within the framework of the project “GWAS of psoriatic arthritis in the Italian population”.

Genomic DNA was extracted from blood samples via a Salting Out modified protocol, quantified with the Quant-iT dsDNA Broad-Range Assay Kit (Invitrogen Life Technologies, Carlsbad, CA, USA) and 200 ng were genotyped for 542,585 genetic markers with the Illumina (San Diego, CA, USA) CoreExomeChip v.1.1 array, which includes the core set of ~280,000 genome-wide SNPs usually implemented in Illumina genotyping arrays, ~250,000 exomic markers and ~20,000 disease-associated variants. MALDI-TOF mass spectrometry-based target genotyping was then used to replicate disease-associated rs1446585 and rs6723108 on 380 additional samples selected according to the above-mentioned criteria to be representative of the examined geographical macro-areas (Supplementary Table S1).

### Data Curation

Analyses described in this study were restricted to 524,738 autosomal loci showing genotyping success rate higher than 95% and no significant HWE deviations (p > 1.9 × 10^−8^ after Bonferroni correction). More stringent per-individual QC procedures were applied as described in Supplementary Information. Despite the sampling campaign was focused on individuals unrelated by at least three generations, cryptic genetic relatedness among subjects was tested by estimating degree of recent shared ancestry (IBD) for each pair of individuals. For this purpose, genome-wide proportion of alleles shared at genotyped loci (i.e. identity by state, IBS) was calculated on a LD-pruned dataset obtained by excluding variants in LD (Supplementary Information). One sample from each pair showing IBD kinship coefficient higher than 0.125 (i.e. third-degree relatives) was removed (n = 21), so that information on 135,905 LD-pruned SNPs typed on 747 individuals were used for subsequent PCA analysis on the Italian dataset.

Such dataset was further filtered for outlier Italian individuals (see PCA and Procrustes Analyses section) and combined with publicly available genome-wide data from 50 populations (Supplementary Information and Supplementary Table S1). According to these procedures, a dataset made up of 55,029 LD-pruned SNPs typed on 737 Italian and 1,282 European/Mediterranean samples was obtained. All QC and merging procedures were carried out by means of the PLINK package.

### Population Structure Analyses

PCA was applied on both the Italian and extended datasets using the R *adegenet* package and according to filtering procedures described in Supplementary Information. To further explore the observed PCA clustering pattern, individuals’ coordinates related to the most informative PCs were averaged within sampling provinces and projected from the PCA space onto their geographic coordinates via Procrustes analysis^50^ performed with the R *vegan* package (Supplementary Information). Resemblance between patterns of genomic and geographic structure was quantified by calculating the t_0_ similarity score and statistical significance was tested with 100,000 permutations. To test robustness of Italian population clusters suggested by PCA and Procrustes analyses, posterior membership probabilities for each individual to belong to a given cluster were calculated and averaged per sampling location via DAPC using the R *adegenet* package and as detailed in Supplementary Information.

### Admixture Analyses

The extended LD-pruned dataset was used to investigate genomic relationships among Italian subpopulations, as well as to confirm possible events of gene flow by constructing maximum likelihood trees with the TreeMix software^51^ (Supplementary Information). The same procedure was applied to the extended dataset including Yoruba as outgroup to inform the position of the root and then rooting trees in subsequent runs using the Beduin sample.

To formally test robustness of observed admixture events, *f3* statistic^52^ was computed for all possible population trios by considering in turn each sample as the result of admixture between all couples of the remaining populations. Z-scores were computed via a Block Jack-knife approach to assess test significance and top 1% of trios ranked according to the lowest Z-score were submitted to further validation using the LD-based approach implemented in ALDER^53^. This enabled to infer also the number of generations since admixture and related time estimates were obtained by assuming 25 years per generation.

Estimates of ancestry proportions were finally obtained using the ADMIXTURE unsupervised clustering algorithm^54^. Probabilistic assignment of each individual to K = 2 through K = 10 hypothetical ancestral populations was calculated and CV procedure was applied to identify the number of clusters for which the model has the best predictive accuracy. Moreover, obtained ancestry fractions at the best fitting K were averaged per sampling location and submitted to Kriging interpolation to produce maps showing their spatial distribution along the Italian peninsula.

### Detection of Genomic Signatures of Natural Selection

#### Single locus F_st_ calculation

To identify genomic regions driving the observed Italian population structure, single locus Weir and Cockerham F_st_ index was computed with the R *pegas* package for each pairwise comparison between population clusters made up of genetically homogeneous provinces. To account for F_st_ dependency on allele frequency and to avoid underestimate of loci characterized by moderate values, but representing significant outliers among those belonging to the same frequency interval, 333,691 variants polymorphic in the examined Italian populations were grouped into bins according to their overall MAF and ranked within each bin on the base of their F_st_ value. Loci scoring in top 1% of F_st_ distribution in each frequency bin were retained as the most differentiated markers.

#### Enrichment Analysis

Enrichment of specific GO terms in genomic regions related to top 1% F_st_ candidates was tested using the GOrilla tool^55^ to pinpoint the most relevant functional pathways involved in differentiation of Italian population clusters. Lists of top candidate genes were compared to the background set of all genes covered by the CoreExomeChip v.1.1 array. Obtain exact p-values were subsequently corrected for multiple testing by controlling false discovery rate (FDR) according to Benjamini and Hochberg procedure. Significant GO terms were further shortlisted into representative, non-redundant subsets using the clustering algorithm based on semantic similarity measures implemented in the REVIGO tool^56^, by focusing on highly specific terms characterized by extremely low proportions (i.e. ≤1%) in the used annotation database.

#### Integrated Haplotype Score Analysis

To investigate potential adaptive events occurred along the Italian peninsula chromosomes of 737 individuals were phased with SHAPEIT2^57^ and the iHS score^41^ was computed for population clusters of genetically homogeneous provinces for each variant showing ancestral allele annotated in the dbSNP database using the R *REHH* package. iHS values were standardized using bins of 10% allele frequency and SNPs showing DAF < 0.2 were excluded to reduce false positives due to the limited power to detect selection at loci with reduced allele frequencies^58^. Rather than focusing on single extreme iHS values, a conservative sliding window approach was applied on a final dataset including 200,714 variants to identify 200 Kb genomic intervals showing the highest fractions of outlier SNPs scoring in top 1% of both F_st_ and |iHS| distributions, as described in Deschamps *et al.*^59^ and in Supplementary Information.

#### Derived Allele Frequency Comparison

To further shortlist potentially adaptive variants characterized by extremely unusual differentiation along the Italian peninsula, top 1% of F_st_ candidates were filtered for genome-wide significant ∆DAF (Supplementary Information). Fisher Exact test was first used to assess significance of ∆DAF between compared population clusters and Bonferroni correction was applied to account for the adopted multiple testing procedures. However, this tested a null hypothesis of no differentiation between the considered populations, assuming that neither genetic drift nor natural selection acted on them. Therefore, correction for background differentiation between Italian population clusters was also applied, as described in Bhatia *et al.*^60^, to test whether ∆DAF at identified candidate loci was due only to genetic drift.

## Additional Information

**How to cite this article**: Sazzini, M. *et al.* Complex interplay between neutral and adaptive evolution shaped differential genomic background and disease susceptibility along the Italian peninsula. *Sci. Rep.* **6**, 32513; doi: 10.1038/srep32513 (2016).

## Supplementary Material

Supplementary Information

Supplementary Tables S2-s3

Supplementary Table S4

Supplementary Dataset

## Figures and Tables

**Table 1:** Loci showing significant ∆DAF differences in N_ITA and S_ITA comparison.

SNP	Chr	Position	S_ITA	N_ITA	∆DAF	Fisher p-val	Corrected p-val	Gene	Type
rs6723108	2	135479980	0.038	0.202	0.164	5.43E-10	1.14E-04	*near TMEM163*	I
rs7570971	2	135837906	0.080	0.275	0.195	6.343E-10	9.57E-05	*RAB3GAP1*	Si
rs17261772	2	135911422	0.234	0.462	0.228	1.161E-08	2.53E-04	*RAB3GAP1*	Sy
rs1446585	2	136407479	0.122	0.336	0.214	7.285E-10	9.89E-05	*R3HDM1*	M

Chr, chromosome; S_ITA, derived allele frequency in S_ITA; N_ITA, derived allele frequency in N_ITA; ∆DAF, difference in derived allele frequency; Fisher p-val, Fisher Exact Test’ s p-values significant after Bonferroni correction; Corrected p-val, p-values calculated by correcting for background differentiation between population clusters; I, intergenic; Si, silent; Sy, synonymous; M, missense.
