# Supplementary material for: Complex interplay between neutral and adaptive evolution shaped differential genomic background and disease susceptibility along the Italian peninsula
Source: Sci Rep. 2016 Sep 1;6:32513. doi: 10.1038/srep32513 (PMC5007512; doi:10.1038/srep32513)
Supplement: Supplementary Information [file srep32513-s1.doc]

**Complex interplay between neutral and adaptive evolution shaped differential genomic background and disease susceptibility along the Italian peninsula**

Marco Sazzini^1^,**^#^,**^*^, Guido Alberto Gnecchi Ruscone^1^,**^#^**, Cristina Giuliani^1^, Stefania Sarno^1^, Andrea Quagliariello^1^, Sara De Fanti^1^, Alessio Boattini^1^, Davide Gentilini^2^, Giovanni Fiorito^3^, Mariagrazia Catanoso^4^, Luigi Boiardi^4^, Stefania Croci^5^, Pierluigi Macchioni^4^, Vilma Mantovani^6^, Anna Maria Di Blasio^2^, Giuseppe Matullo^3^, Carlo Salvarani^4^, Claudio Franceschi^7^, Davide Pettener^1^; Paolo Garagnani^7,†^ & Donata Luiselli^1,†^

^1^Laboratory of Molecular Anthropology and Centre for Genome Biology, Department of Biological, Geological and Environmental Sciences, University of Bologna, Bologna, Italy.

^2^Centre for Biomedical Research and Technologies, Italian Auxologic Institute, IRCCS, Milano, Italy.

^3^Department of Medical Sciences, University of Torino and Human Genetics Foundation, Torino, Italy.

^4^Rheumatology Unit, Arcispedale Santa Maria Nuova Hospital, IRCCS, Reggio Emilia, Italy.

^5^Allergy and Advanced Biotechnologies Unit, Arcispedale Santa Maria Nuova Hospital, IRCCS, Reggio Emilia, Italy.

^6^Center for Applied Biomedical Research, S. Orsola-Malpighi University Hospital, Bologna, Italy.

^7^Department of Experimental, Diagnostic and Specialty Medicine, University of Bologna, Bologna, Italy.

^*^corresponding author (email: [marco.sazzini2@unibo.it](mailto:marco.sazzini2@unibo.it))

**^#^,**^†^These authors contributed equally to this work

**Supplementary Information**

Includes Supplementary Figures S1-S7, Supplementary Table S1, Supplementary Results and Discussion, Supplementary Methods, Supplementary References.

**Supplementary Figures**

**Supplementary Fig. S1.** **First and second PCs computed on the Italian dataset.** Outlier individuals with respect to the bulk of samples collected in their province of origin were identified according to eigenvectors for one of the displayed PCs exceeding ± 3 standard deviations from the mean calculated for that sampling location and were removed from subsequent analyses.


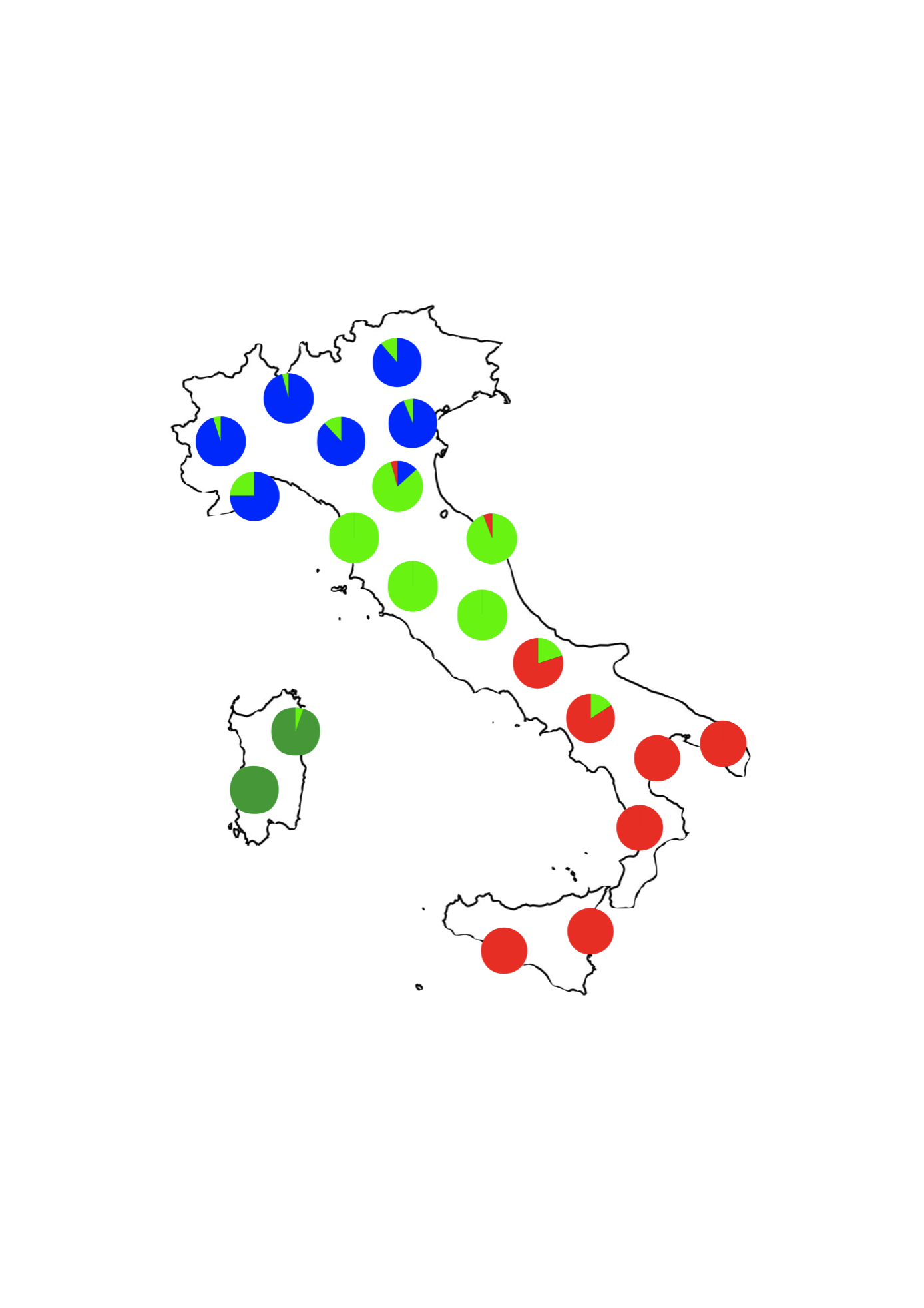


**Supplementary Fig. S2.** **Individual posterior membership probabilities to belong to population clusters suggested by PCA and Procrustes analyses calculated via DAPC and averaged per sampling location.** Provinces clustering within the N_ITA, C_ITA, S_ITA and SARD groups are displayed in blue, green, red, and dark green, respectively. DAPC analysis and map of the Italian peninsula were plotted using the R software v.3.1.1 (R: A Language and Environment for Statistical Computing, R Core Team, R Foundation for Statistical Computing, Vienna, Austria (2016) [https://www.R-project.org](https://www.R-project.org/)).

**Supplementary Fig. S3.** **Matrix of residuals associated to TreeMix analysis performed on the Italian dataset.** The related tree was constructed by not considering migration in order to evaluate the overall topology and the extent of drift in terms of allele frequency shift from an ancestral population.


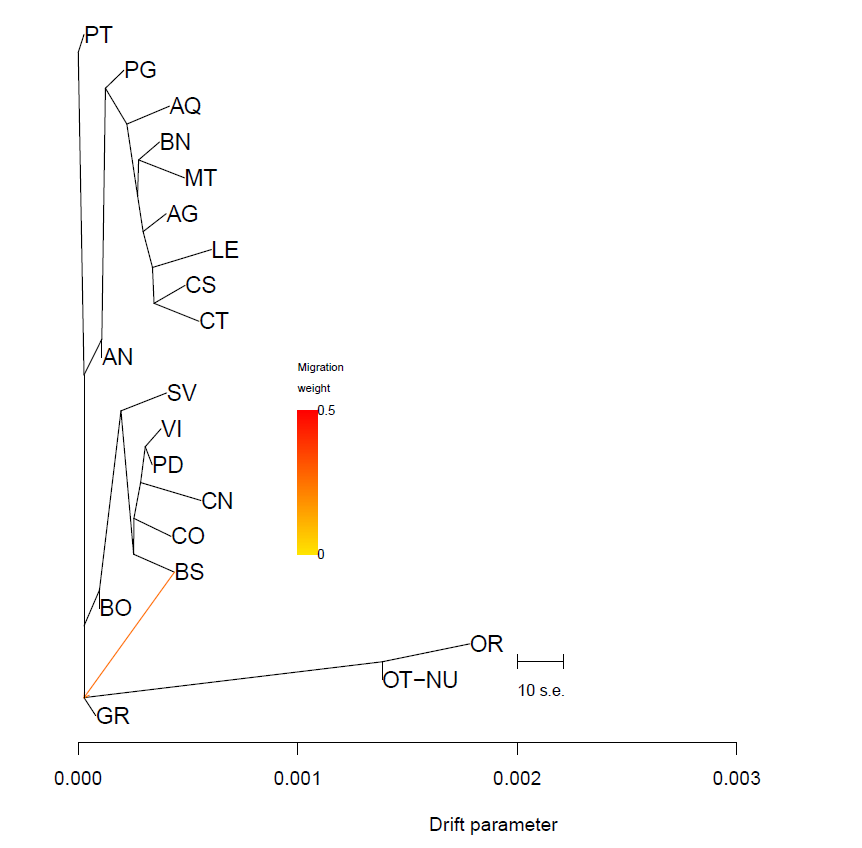

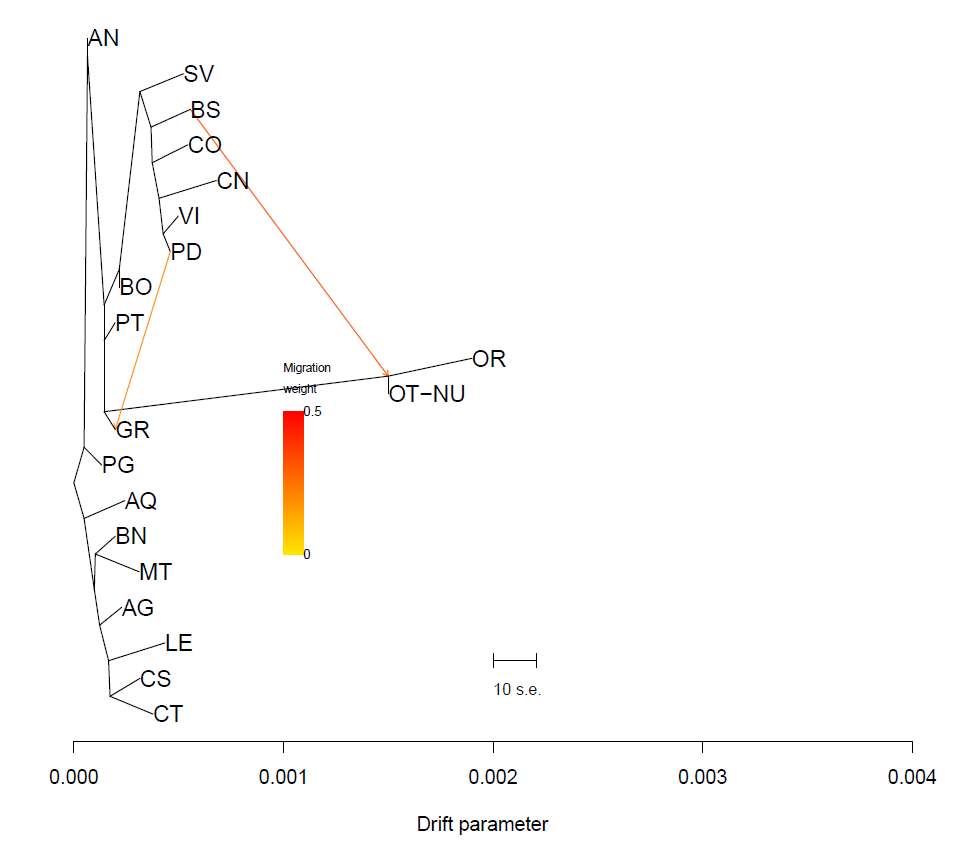


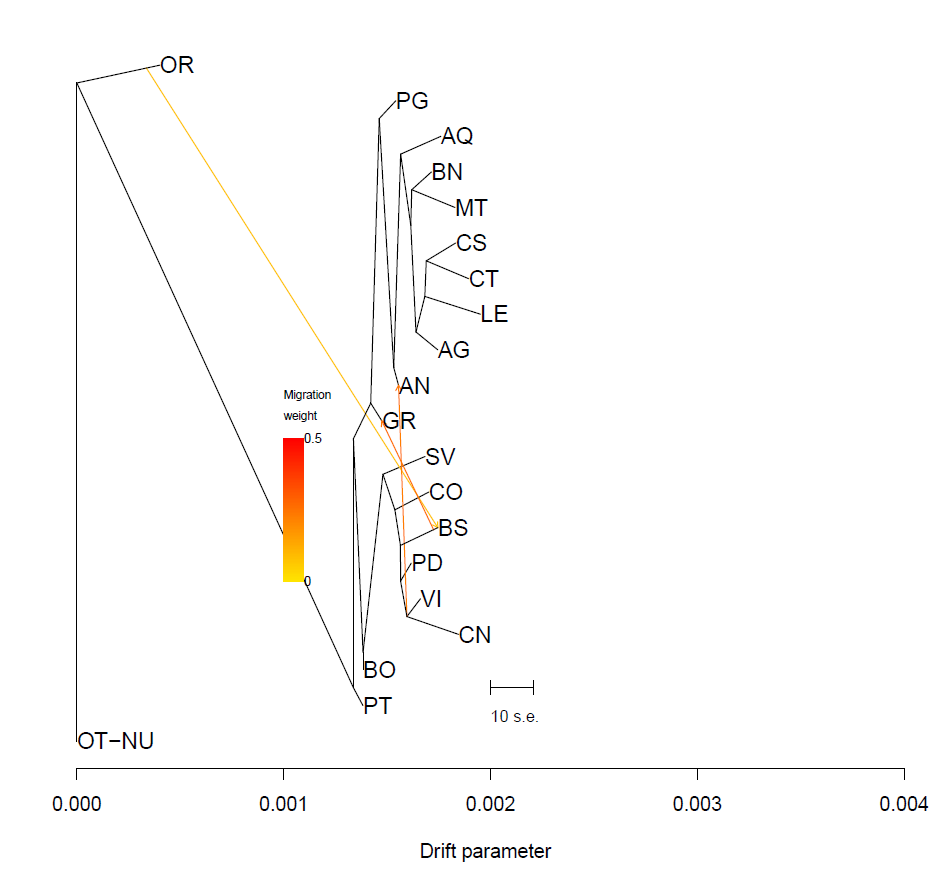

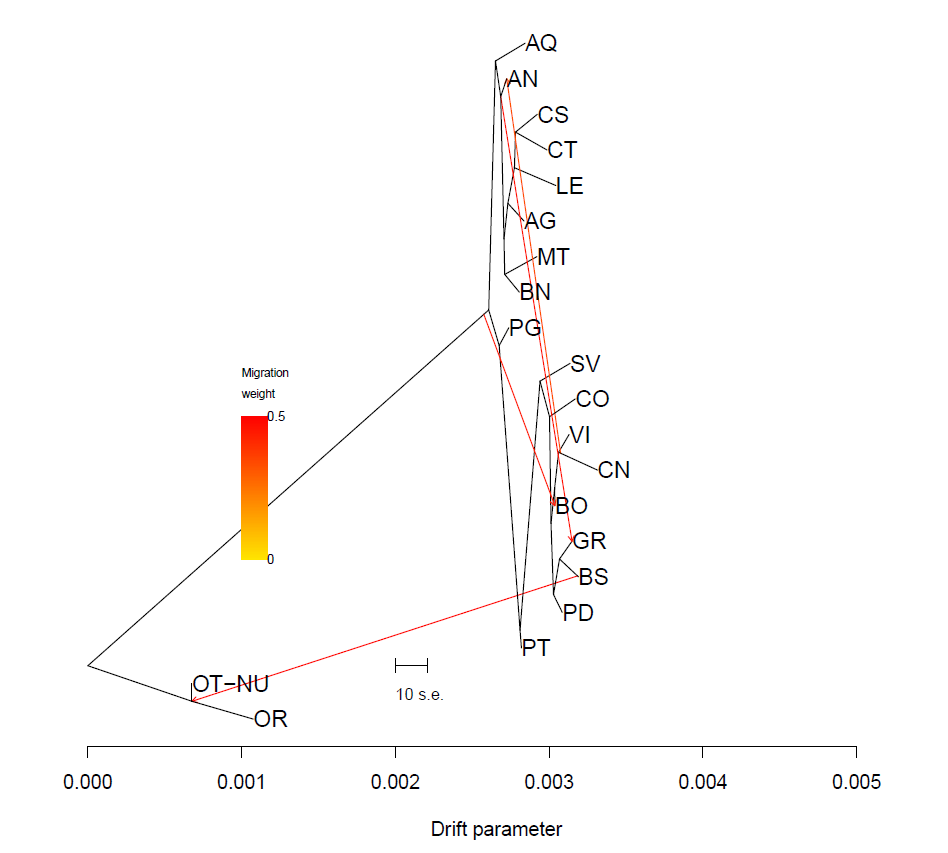


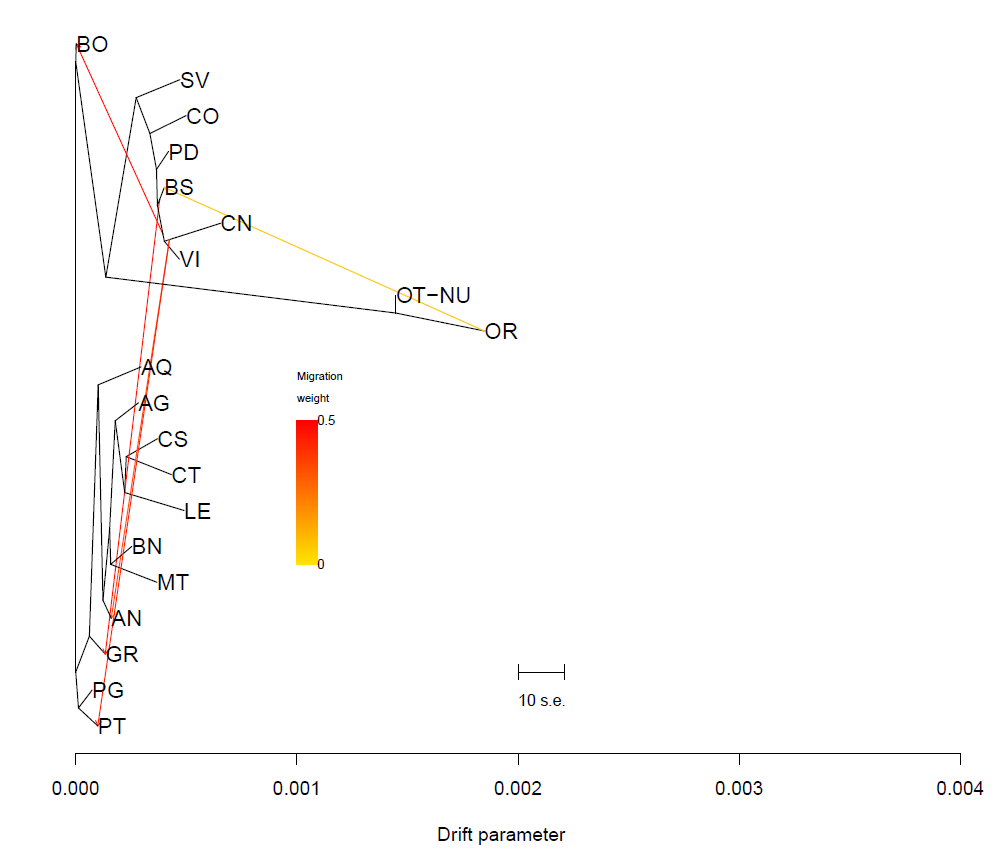

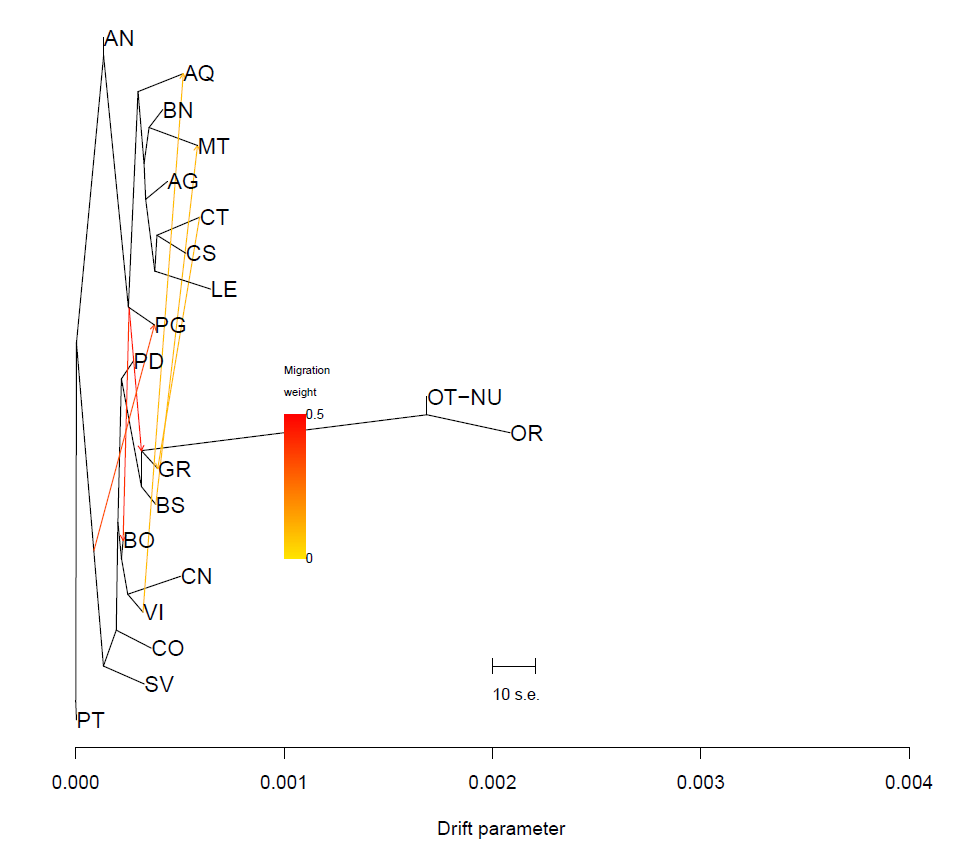


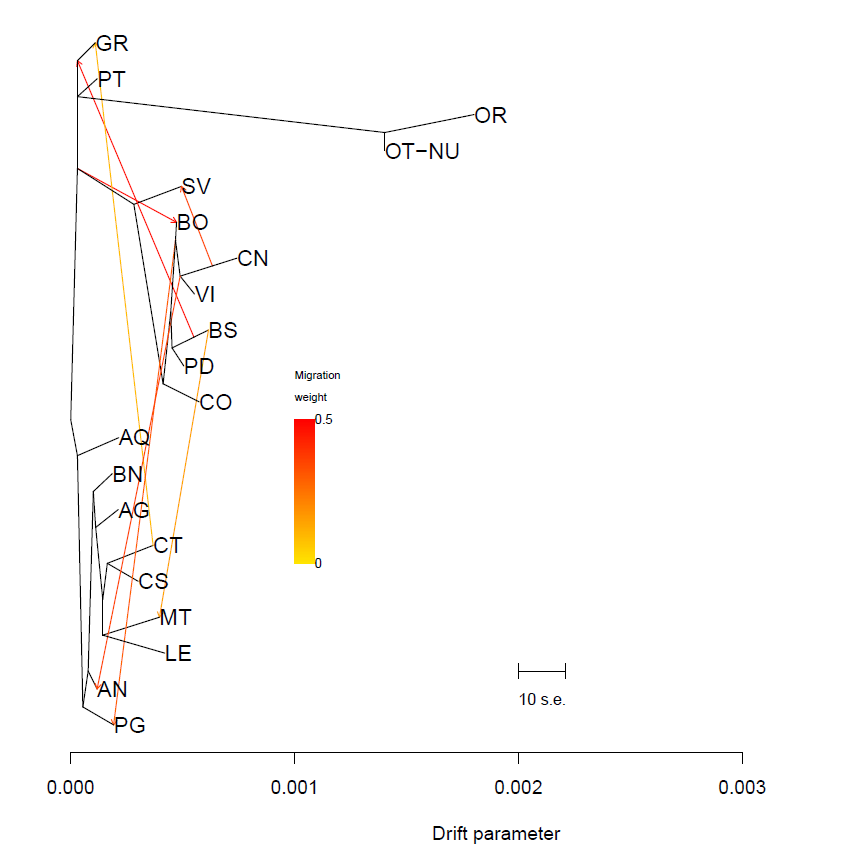


**Supplementary Fig. S4.** **TreeMix graphs describing the splitting patterns observed in the Italian dataset.** Trees were constructed by allowing for 1-7 migration edges (m, from left to right), with m = 6 and m = 7 resolving most of the population pairs with poor fits of the model to the data according to analysis of the matrices of residuals. The length of the branches is proportional to the genetic drift experienced by each population.

**
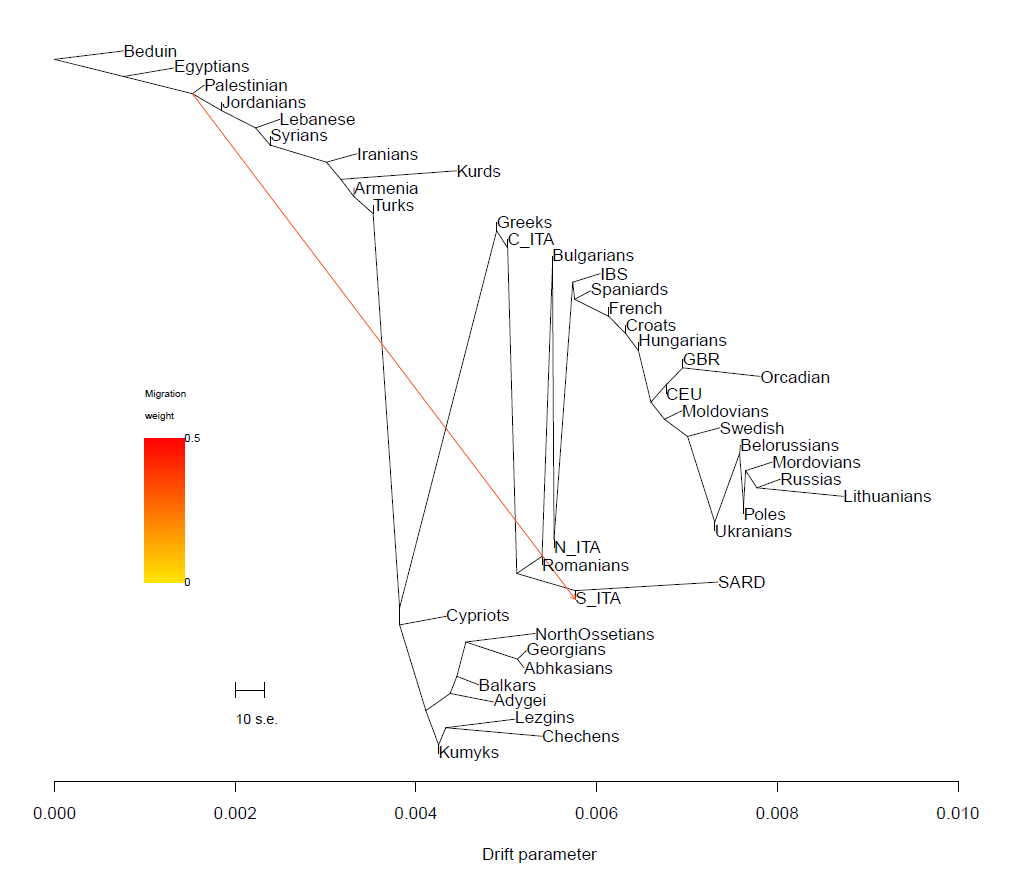
**

**
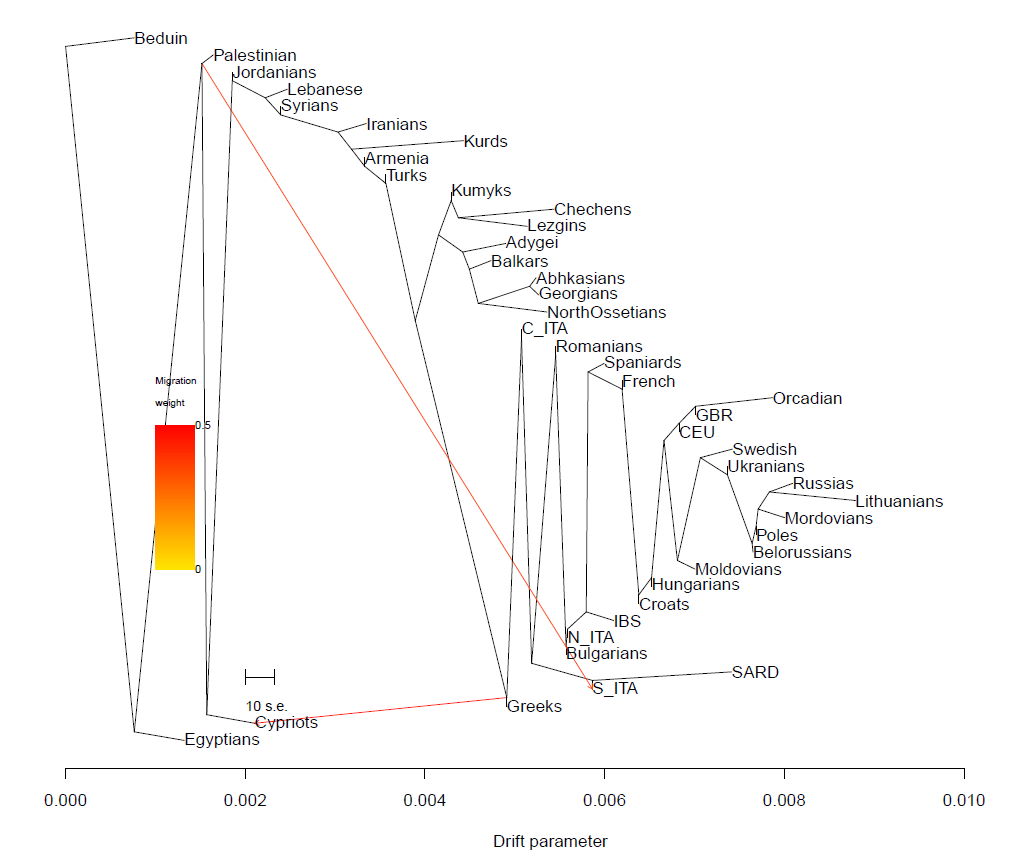
**

**
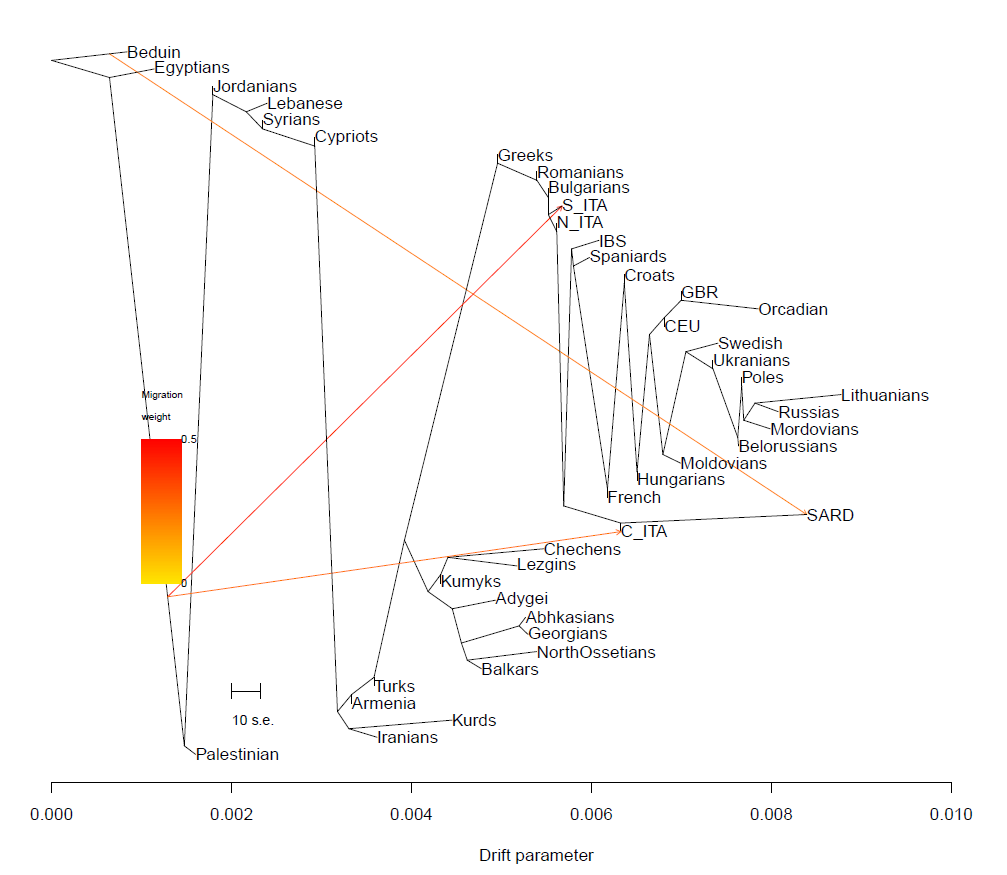
**


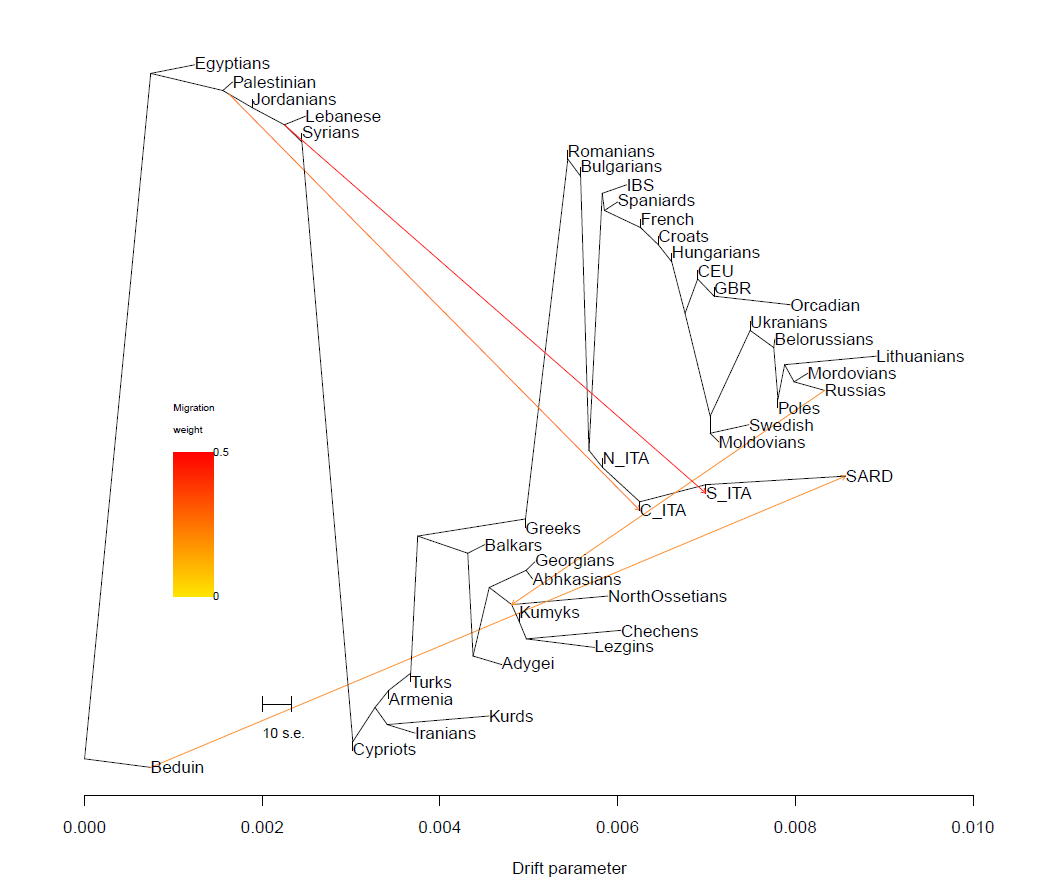


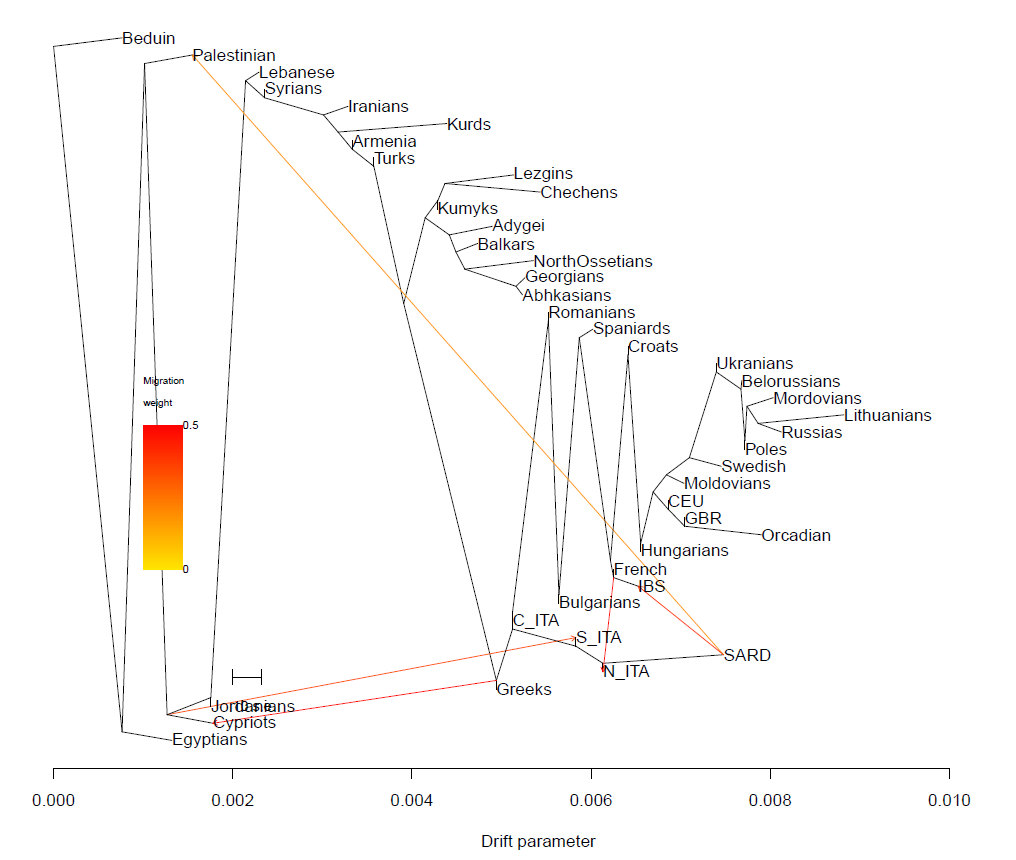


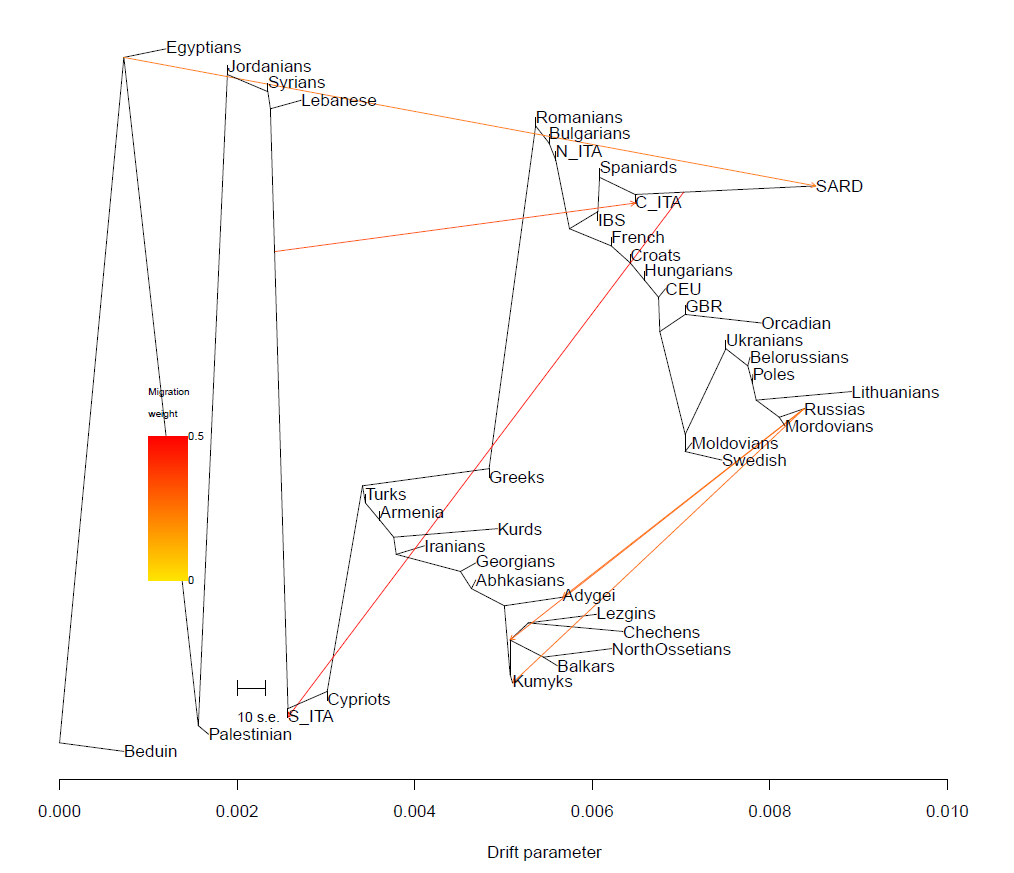


**Supplementary Fig. S5.** **TreeMix graphs describing the splitting patterns observed in the extended dataset.** Trees were constructed by allowing for 1-6 migration edges (m, from top to bottom), with m = 6 minimizing population pairs with poor fits of the model to the data according to analysis of the matrices of residuals. The length of the branches is proportional to the genetic drift experienced by each population.

**
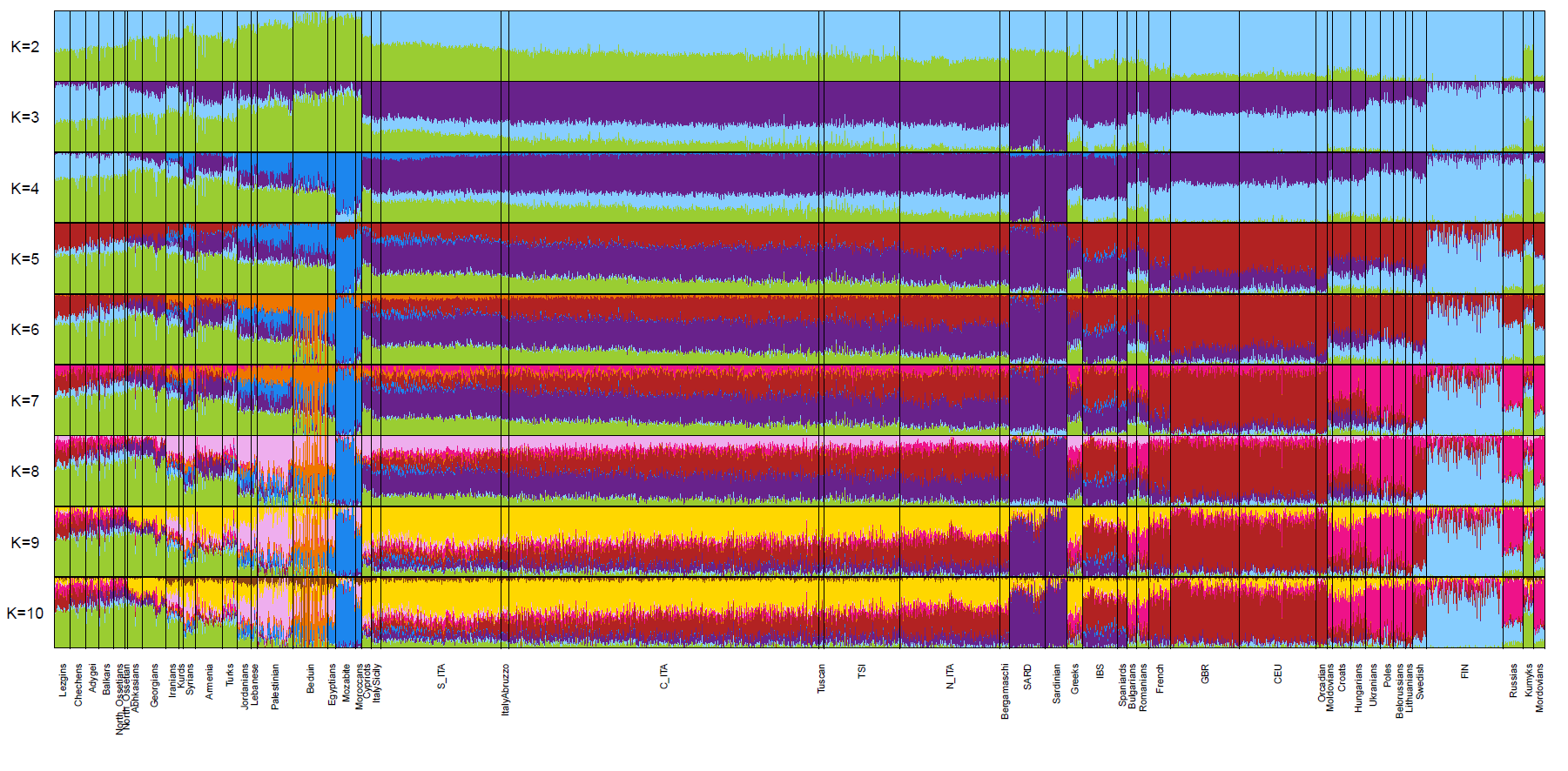
**

**Supplementary Fig. S6.** **Ancestry proportions at K = 2 to K = 10 estimated by means of ADMIXTURE clustering analysis performed on the extended dataset**. The analysed dataset included 2,019 individuals belonging to the studied Italian population clusters and to 50 Mediterranean and European human groups.

**Supplementary Fig. S7.** **Cross-validation errors of ADMIXTURE clustering analysis performed on the extended dataset.** The best predictive accuracy was achieved by the model when four or five ancestral populations (K = 4 or K = 5) were hypothesized.

**Supplementary Tables**

**Supplementary Table S1.** Italian samples typed in the present study and European/Mediterranean populations included in the reference dataset.

| **Population** | **Acronym** | **Country** | **Macro Area** | **Source** | **SNP Chip** | **N** |
| --- | --- | --- | --- | --- | --- | --- |
| Cuneo | CN | Italy | Europe | present study | Illumina CoreExome | 20 |
| Savona | SV | Italy | Europe | present study | Illumina CoreExome | 20 |
| Savona | SV | Italy | Europe | present study | Sequenom replic. | 10 |
| Imperia | IM | Italy | Europe | present study | Sequenom replic. | 27 |
| Como | CO | Italy | Europe | present study | Illumina CoreExome | 24 |
| Varese | VA | Italy | Europe | present study | Sequenom replic. | 26 |
| Brescia | BS | Italy | Europe | present study | Illumina CoreExome | 25 |
| Vicenza | VI | Italy | Europe | present study | Illumina CoreExome | 26 |
| Vicenza | VI | Italy | Europe | present study | Sequenom replic. | 10 |
| Trieste | TS | Italy | Europe | present study | Sequenom replic. | 36 |
| Padova | PD | Italy | Europe | present study | Illumina CoreExome | 16 |
| Belluno | BL | Italy | Europe | present study | Sequenom replic. | 26 |
| Bologna | BO | Italy | Europe | present study | Illumina CoreExome | 208 |
| Pistoia | PT | Italy | Europe | present study | Illumina CoreExome | 11 |
| Grosseto | GR | Italy | Europe | present study | Illumina CoreExome | 28 |
| Grosseto | GR | Italy | Europe | present study | Sequenom replic. | 10 |
| Foligno/PG | PG | Italy | Europe | present study | Illumina CoreExome | 24 |
| Foligno/PG | PG | Italy | Europe | present study | Sequenom replic. | 10 |
| Terni | TR | Italy | Europe | present study | Sequenom replic. | 30 |
| Pesaro | PU | Italy | Europe | present study | Sequenom replic. | 20 |
| Ancona | AN | Italy | Europe | present study | Illumina CoreExome | 141 |
| Macerata | MC | Italy | Europe | present study | Sequenom replic. | 26 |
| Ascoli Piceno | AP | Italy | Europe | present study | Sequenom replic. | 20 |
| L'Aquila | AQ | Italy | Europe | present study | Illumina CoreExome | 20 |
| Benevento | BN | Italy | Europe | present study | Illumina CoreExome | 19 |
| Benevento | BN | Italy | Europe | present study | Sequenom replic. | 10 |
| Campobasso | CB | Italy | Europe | present study | Sequenom replic. | 25 |
| Matera | MT | Italy | Europe | present study | Illumina CoreExome | 20 |
| Matera | MT | Italy | Europe | present study | Sequenom replic. | 15 |
| Lecce | LE | Italy | Europe | present study | Illumina CoreExome | 20 |
| Lecce | LE | Italy | Europe | present study | Sequenom replic. | 10 |
| Cosenza | CS | Italy | Europe | present study | Illumina CoreExome | 19 |
| Cosenza | CS | Italy | Europe | present study | Sequenom replic. | 10 |
| Catanzaro | CZ | Italy | Europe | present study | Sequenom replic. | 10 |
| Reggio Calabria | RC | Italy | Europe | present study | Sequenom replic. | 26 |
| Catania | CT | Italy | Europe | present study | Illumina CoreExome | 29 |
| Enna | EN | Italy | Europe | present study | Sequenom replic. | 23 |
| Agrigento | AG | Italy | Europe | present study | Illumina CoreExome | 30 |
| Oristano | OR | Italy | Europe | present study | Illumina CoreExome | 28 |
| OlbiaTempio/Nuoro | OT-NU | Italy | Europe | present study | Illumina CoreExome | 19 |
| Abhkasians | Abh | Abkhazia (Georgia) | Caucasus | Yunusbayev et al 2011 | Illumina 610K | 20 |
| Adygei | Ady | North Caucasus | Russia | Li et al 2008 | Illumina 660k | 17 |
| Armenians | Ar | Armenia | Caucasus | Behar et al 2010 | Illumina 660k | 35 |
| Balkars | Bal | Russia (Kabardino-Balkaria) | Caucasus | Yunusbayev et al 2011 | Illumina 610K | 19 |
| Beduins | Bed | Israel-Negev | Middle East | Li et al 2008 | Illumina 660k | 45 |
| Belorussians | Bel | Belorussia | Europe | Behar et al 2010 | Illumina 660k | 17 |
| Italians Bergamo | Ber_ITA | Italy (Bergamo) | Europe | Li et al 2008 | Illumina 660k | 12 |
| Bulgarians | Bul | Bulgaria | Europe | Yunusbayev et al 2011 | Illumina 610K | 13 |
| CEU | CEU | Utah residents (N/W European ancestry) | USA | 1000 Genomes Project | Whole genome seq | 104 |
| Chechens | Chec | Russia (Chechnya) | Caucasus | Yunusbayev et al 2011 | Illumina 610K | 20 |
| Chuvashs | Chu | Russia | Europe | Behar et al 2010 | Illumina 660k | 17 |
| Croats | Cr | Croatia | Europe | Behar et al 2012 | Illumina Omni 1M | 24 |
| Cypriots | Cyp | Cyprus | Europe | Behar et al 2010 | Illumina 660k | 12 |
| Egyptians | Egy | Egypt | North Africa | Behar et al 2010 | Illumina 660k | 12 |
| FIN | FIN | Finland | Europe | 1000 Genomes Project | Whole genome seq | 99 |
| French | Fr | France | Europe | Li et al 2008 | Illumina 660k | 28 |
| GBR | GBR | Great Britain | Europe | 1000 Genomes Project | Whole genome seq | 95 |
| Georgians | Geo | Georgia | Caucasus | Behar et al 2010 | Illumina 660k | 30 |
| Greeks | Gre | Greece | Europe | Behar et al 2012 | Illumina Omni 1M | 20 |
| Hungarians | Hun | Hungary | Europe | Behar et al 2010 | Illumina 660k | 20 |
| IBS | IBS | Iberia | Europe | 1000 Genomes Project | Whole genome seq | 46 |
| Iranians | Ira | Iran | Middle East | Behar et al 2010 | Illumina 660k | 20 |
| Italians Abruzzo | Ab_ITA | Italy | Europe | Behar et al 2012 | Illumina Omni 1M | 11 |
| Italians Sicily | Sic_ITA | Italy | Europe | Behar et al 2012 | Illumina Omni 1M | 13 |
| Jordanians | Jor | Jordania | Middle East | Behar et al 2010 | Illumina 660k | 20 |
| Kumyks | Kum | Russia (Kumyk plateau) | Caucasus | Yunusbayev et al 2011 | Illumina 610K | 14 |
| Kurds | Kur | Kurdistan | Middle East | Yunusbayev et al 2011 | Illumina 610K | 6 |
| Lebanese | Leb | Lebanon | Middle East | Behar et al 2010 | Illumina 660k | 7 |
| Lezgins | Lez | Russia (Dagestan) | Caucasus | Behar et al 2010 | Illumina 660k | 21 |
| Lithuanians | Lit | Lithuania | Europe | Behar et al 2010 | Illumina 660k | 10 |
| Moldovians | Mol | Moldavia | Europe | Behar et al 2012 | Illumina Omni 1M | 7 |
| Mordovians | Mor | Russia | Europe | Yunusbayev et al 2011 | Illumina 610K | 15 |
| Moroccans | Mo | Morocco | North Africa | Behar et al 2010 | Illumina 660k | 10 |
| Mozabites | Moz | Algeria-Mzab | North Africa | Li et al 2008 | Illumina 660k | 27 |
| Nogais | Nog | Russia (Dagestan) | Caucasus | Yunusbayev et al 2011 | Illumina 610K | 16 |
| North Ossetians | NO | Russia (North Ossentia) | Caucasus | Behar et al 2012 | Illumina Omni 1M | 3 |
| North Ossetians | NO | Russia (North Ossentia) | Caucasus | Yunusbayev et al 2011 | Illumina 610K | 15 |
| Orcadians | Or | OrkneyIslands | Europe | Li et al 2008 | Illumina 660k | 15 |
| Palestinians | Pal | Israel-Central | Middle East | Li et al 2008 | Illumina 660k | 46 |
| Palestinians | Pal | Israel | Middle East | Behar et al 2012 | Illumina Omni 1M | 6 |
| Poles | Pol | Poland | Europe | Behar et al 2012 | Illumina Omni 1M | 17 |
| Romanians | Rom | Romania | Europe | Behar et al 2010 | Illumina 660k | 16 |
| Russians | Ru | Russia | Europe | Behar et al 2010 | Illumina 660k | 2 |
| Russians | Ru | NorthWestern Russia | Europe | Li et al 2008 | Illumina 660k | 25 |
| Sardinians | Sar_ITA | Italy (Sardinia) | Europe | Li et al 2008 | Illumina 660k | 28 |
| Spaniards | Spa | Spain | Europe | Behar et al 2010 | Illumina 660k | 12 |
| Swedish | Swe | Sweden | Europe | Behar et al 2012 | Illumina Omni 1M | 18 |
| Syrians | Sy | Syria | Middle East | Behar et al 2010 | Illumina 660k | 16 |
| TSI | TSI | Italy | Europe | 1000 Genomes Project | Whole genome seq | 100 |
| Turks | Tur | Turkey | Near East | Behar et al 2010 | Illumina 660k | 19 |
| Tuscans | Tu_ITA | Italy (Tuscany) | Europe | Li et al 2008 | Illumina 660k | 7 |
| Ukranians | Ukr | Ukraine | Europe | Yunusbayev et al 2011 | Illumina 610K | 20 |
| Uzbeks | Uz | Uzbekstan | Central Asia | Behar et al 2010 | Illumina 660k | 15 |

*Sequenom replic.*, samples included in the panel used for replication with MALDI-TOF mass spectrometry-based target genotyping on Sequenom platform.

Supplementary Tables S2-S6 are included in three Excel files.

**Supplementary Table S2.** Top 1% of f3 values according to computed Z-scores (on the left) and significant population trios retained after ALDER analysis (on the right).

**Supplementary Table S3.** Genes covered by top 1% Fst SNPs in the performed pairwise population comparisons.

**Supplementary Table S4.** Enriched Gene Ontology (GO) terms in the performed pairwise population comparisons.

**Supplementary Table S5.** Genomic intervals scoring in top 0.1% of windows with the highest fractions of F_st_ and |iHS| outlier SNPs.

**Supplementary Table S6.** Replication on an independent Italian dataset of iHS analysis for the top candidate genomic regions identified in the present study.

**Supplementary Results and Discussion**

**Fine Dissection of the Italian Population Structure**

A first explorative PCA was performed after having ruled out first-, second- or third-degree kinship of the examined subjects according to the estimate of genome-wide IBD proportion at the genotyped loci. Outlier positions in the genomic space depicted by PCA were appreciated for 10 subjects with respect to the bulk of samples collected at their province of origin, guiding a further refinement of the studied dataset prior to subsequent analyses. Intersection between samples from diverse macro-areas was limited to a subset of individuals from Bologna (C_ITA), which showed close affinity to those from northern provinces, such as Savona, Padova and Vicenza, while some subjects from Ancona (C_ITA), as well as most from L’Aquila (C_ITA), actually clustered within the bulk of southern samples.

An explorative PCA including 50 additional European and Mediterranean human groups for which genotype data were available from literature (Supplementary Table S1) pointed to Chuvash, Nogai and Uzbek samples as genetic outliers. After their exclusion, N_ITA samples overlapped with Northern Italians available from literature and, partially, with some Spanish and French groups, as well as with a limited number of C_ITA individuals. People from C_ITA instead appeared to be more scattered along PC2, overlapping with previously typed Tuscan samples, but also showing subjects that grouped within the N_ITA or S_ITA populations. This latter group absorbed Sicilian individuals from literature and was appreciably homogeneous, similarly to the SARD one, which overlapped with Sardinians from databases, but with the exception of few subjects occupying an intermediate position between SARD and C_ITA samples (Fig. 2a).

Analysis of residuals obtained via Procrustes test (Fig. 1) revealed that the two SARD samples showed the highest residual values (0.34 and 0.30 for the provinces of Oristano and Olbia-Tempio/Nuoro, respectively) confirming that the well-known genomic divergence between Sardinians and peninsular Italians is considerably higher than that expected according to their geographic distance, in accordance with the evidence of high drift occurred in the Sardinian groups due to long-term isolation provided by several studies^1-4^. The remaining highest residuals were computed for samples from Cuneo (0.16) and Savona (0.13), as well as from Agrigento (0.17), Catania (0.12) and Lecce (0.12).

When averaged per sampling location, membership probabilities computed via DAPC (Supplementary Fig. S2) showed a high percentage (95%) of Central Italians actually assigned to C_ITA, but only by considering subjects from L’Aquila as belonging to the S_ITA group. The overall 5% of C_ITA miss-assigned samples was instead due some individuals from Bologna that were predicted to belong to the N_ITA cluster and few ones to S_ITA (13% and 4%, respectively), the latter case being observed also for 6% of people from Ancona. Such genetic homogeneity turned out to be comparable also within the S_ITA cluster, with subjects of more plausible C_ITA affiliation being limited to the provinces of L’Aquila and Benevento (20% and 16%, respectively), and was even higher in Sardinia. In fact, only 3% of the examined SARD individuals showed membership probabilities in line with their grouping to the C_ITA cluster, all of them being restricted to the province of Olbia-Tempio/Nuoro. Conversely, an appreciable genetic continuity between northern and central Italian populations was observed according to the 11% of northern individuals being assigned to the C_ITA group. Moreover, all northern provinces showed a fraction of subjects genetically closer to people from C_ITA than from N_ITA, with the sample from Savona reaching the highest proportion (25%).

TreeMix analyses indicated that despite the identification of two well-defined N_ITA and S_ITA clades, the sample from Savona could be appreciably distinguished from the bulk of N_ITA provinces, as already suggested by DAPC. Moreover, people from L’Aquila, clustered in the S_ITA clade, branching out after the split between the Ancona and Perugia samples (C_ITA), in proximity to the latter (Fig. 2b and Supplementary Fig. S3). The first three TreeMix runs allowing for increasing migration edges (m) involved the province of Brescia and the node of SARD (m = 1) or the ancestral population from which the two SARD samples split (m = 2-3), as well as some provinces belonging to the N_ITA and C_ITA clusters (i.e. Padova and Grosseto, m = 2; Brescia and Grosseto, Vicenza and Ancona, m = 3). Further increase in the allowed m added events mainly restricted to C_ITA and N_ITA samples (e.g. Ancona-Grosseto m = 4, Ancona-Cuneo, m = 4-5), while admixture involving also S_ITA provinces appeared only at m = 6 and m = 7 (e.g. L’Aquila-Vicenza, Brescia-Matera, Catania-Grosseto) by resolving most of the population pairs with poor fits of the model to the data pointed out by matrices of residuals (Supplementary Fig. S4).

**Inferring Admixture Events between Italian and European/Mediterranean populations**

When the TreeMix algorithm was applied to the extended dataset, the most recurrent admixture events involved S_ITA and the node of Palestinians (m = 1, m = 2) or a population ancestral to present-day Palestinians (m = 3), as well as the latter and C_ITA (m = 3, m = 4), in addition to SARD and a population ancestral to Beduins (m = 3, m = 4) or Palestinians (m = 5). However, m = 6 actually minimized population pairs with poor fits of the model to the data. Among these six most plausible admixture events, some involved the node of Egyptians and SARD, a population ancestral to Lebanese and S_ITA in relation to C_ITA, as well as a group ancestral to SARD and C_ITA as strongly admixed with S_ITA (Supplementary Fig. S5).

When top 1% of most significant *f3* values were retained according to computed Z-scores, 85% of tested population trios actually involved Italian clusters (Supplementary Table S2). In addition to the pattern described in the main text, the SARD sample seemed to have played a major role as source of admixture for most of the examined populations, especially Italian ones, rather than as recipient of migratory processes. In fact, the most significant *f3* scores for trios including SARD indicated peninsular Italians as plausible results of admixture between SARD and populations from Iran, Caucasus and Russia. This scenario could be interpreted as further evidence that Sardinians retain high proportions of a putative ancestral genomic background that was considerably widespread across Europe at least until the Neolithic and that has been subsequently erased or masked in most of present-day European populations^5^.

Further validation of the inferred events of gene flow was obtained via ALDER analysis, despite it was proved to be extremely sensible to background LD, which is considerably shared between related populations, such as those belonging to the European continent. In fact, it is well known that Europeans exhibit a continuous gradient of genetic variation concordant with their geographic distribution rather than grouping into discrete genetic clusters^6^. Therefore, it is reasonable to expect background LD to be negligible only between populations located at the opposite extremes of such a gradient. For this reason, most of the significant population trios identified according to *f3* computation were not confirmed by ALDER analysis, which instead pinpointed only the most differentiated groups as the best proxies for the possible parental populations involved in admixture events with Italians (Supplementary Table S2). Moreover, by fitting an exponential curve to the decay of admixture-induced LD as a function of genetic distance this approach is able to detect admixture events occurred between a test population and two proxies of the possible ancestral mixing populations. Time estimates obtained by fitting one exponential curve do not necessarily imply that only one admixture event had occurred. In fact, the ALDER algorithm calculates the mean time between all the inferred admixture events weighted by admixture proportions.

**Signatures of Natural Selection on the Italian Genomes**

In addition to significantly enriched GO terms observed in N_ITA and S_ITA comparison, loci encoding for components of extracellular matrix were significantly over-represented also in the N_ITA/C_ITA comparison, while GO terms related to components of membrane raft and axon/synapse were enriched among the genes most differentiated between C_ITA and S_ITA. Moreover, when considering highly divergent loci between SARD and each peninsular population cluster genes encoding for different cellular components were enriched in the diverse comparisons. In detail, when comparing N_ITA and S_ITA clusters, 47% of the top F_st_ candidates were distributed on 910 different genes (Supplementary Table S3). The most over-represented GO terms were associated to biological processes, such as cell or neuron recognition (GO:0008037, adjusted p = 8.8x10^-04^, GO:0008038, adjusted p = 1.4x10^-04^) and regulation of cell projection organization (GO:0030030, adjusted p = 2.4x10^-04^), as well as to cellular components, such as those of extracellular matrix (GO:0044420, adjusted p = 2.1x10^-05^), basement membrane (GO:0005604, adjusted p = 1.7x10^-03^), dendrites (GO:0030425, adjusted p = 2.8x10^-03^) and membrane raft (GO:0045121, adjusted p = 1.8x10^-03^). When N_ITA and C_ITA clusters were contrasted, 53% of top F_st_ candidates lie on the 1,143 most differentiated genes (Supplementary Table S3) and loci encoding for components of extracellular matrix were significantly over-represented (GO:0044420, adjusted p = 2.9x10^-04^). The 1,257 most differentiated genes between C_ITA and S_ITA clusters instead harboured 49% of related top F_st_ candidates (Supplementary Table S3), with GO terms related to cellular components (i.e. membrane raft, GO:0045121, adjusted p = 3.2x10^-05^; axon, GO:0033267, adjusted p = 1.6x10^-03^ and synapse, GO:0044456, adjusted p = 2.8x10^-03^) being the most enriched. Finally, when highly divergent loci were searched by contrasting SARD and peninsular population clusters, genes encoding for cellular components were differentially enriched in the diverse comparisons. In particular, loci associated to the Golgi membrane (GO:0000139, adjusted p = 2.7x10^-03^) were enriched among the 1,331 genes highly differentiated between SARD and C_ITA and accounting for 59% of top F_st_ candidates (Supplementary Table S3). GO terms related to components of synapses (GO:0044456, adjusted p = 1.63x10^-03^) were over-represented in the 1,032 genes on which 51% of top candidates differentiating SARD and N_ITA clusters were located (Supplementary Table S3). Loci encoding for components of the transmembrane transporter complex (GO:1902495, adjusted p=3.5x10^-03^) were instead enriched among the 1,165 genes harbouring 54% of loci highly differentiated between the SARD and S_ITA samples (Supplementary Table S3).

The ten most plausible candidate chromosomal regions to have undergone positive selection in N_ITA involved nine genes and two regulatory sequences (Supplementary Table S5). A putative signature detected at a window including SNPs on the *RP11-236P13.1* novel lincRNA was restricted to N_ITA. Interestingly, an increasing number of studies have proved that lincRNAs plausibly influence expression of both nearby and distantly located genes^7,8^ and *RP11-236P13.1* transcription was recently found to be down regulated in macrophage-derived foam cells characteristic of atherosclerosis and responsible for increased risk of cardiovascular disease^9^. Although the functional impact of the identified candidate variants is unknown, this finding suggests that such a lincRNA might play a role in the maintenance of cholesterol homeostasis. A second N_ITA specific signal emerged at a genomic window encompassed within the *WDPCP* gene, a locus participating to cell projection organization and whose variants modify phenotypic manifestations of Bardet-Biedl syndrome 15 related to adipogenic processes responsible for early-onset obesity^10^. By considering TSI as a rough approximation of the overall Italian population and thus enabling exploitation of whole-genome sequence data, it was possible to verify that rs17408841 included in the *WDPCP* region putatively targeted by selection is in strong LD (r^2^ = 0.97) with a variant (rs13431765) known to have a strong functional effect. In fact, such SNP was proved to modulate levels of C-reactive protein maintaining them in the physiological range even in the context of high dietary intake of trans-unsaturated fatty acids, thus contributing to reduced risk for cardiovascular disease, coronary artery disease (CAD) and type 2 diabetes (T2D) ^11,12^. A further N_ITA specific signature spanning from a CTCF binding site to an enhancer sequence in the *RNMTL1P2* promoter-flanking region was observed. These regulatory regions are active in macrophages and fibroblasts and could modulate the expression of the *RNMTL1P2* processed pseudogene. Two candidate selected SNPs in such a genomic window (rs4543441 and rs11766489) turned out to be in high LD (r^2^ = 0.96 and r^2^ = 0.88, respectively) in TSI with rs10226084, a variant that influences regulation of fat metabolism and that is associated to reduced levels of plasma fibrinogen. Such a rs10226084-mediated phenotypic trait strongly correlates with BMI and high-density lipoprotein cholesterol concentration so that this SNP was proposed to exert a protective role against CAD ^13^.

A total of ten genes and one regulatory region were distributed on ten genomic intervals identified as potential targets of positive selection in C_ITA, none of them resulting restricted to this population group. The most extended signatures emerged at loci involved in B cells functionality. A first one was related to a window covering part of the *DLEU1* lincRNA and its promoter-flanking region and was shared with people from S_ITA (Supplementary Table S5). Due to its deletion in patients affected by B-cell chronic lymphocytic leukemia, *DLEU1* is considered as a tumour suppressor essential for development of an efficient population of B-lymphocytes able to produce the full spectrum of possible antibodies^14^. All putative *DLEU1* selected SNPs are known to be associated to reduced height variation in populations of European ancestry and were proposed as suggestive evidence of a tight link between physiological body growth and processes regulating cell differentiation and immune specialization^15^. A second signal instead encompassed an enhancer sequence mapping near the *TNKS* gene and potentially able to regulate its expression, as well as one of its intron. This signature was limited to the sole enancher sequence in N_ITA, while further extended of additional 50kb upstream of the gene in S_ITA. In TSI, the putative non-selected allele observed at the *TNKS* enhancer rs4841254 is in high LD (r^2^ = 0.91) with the rs2055729 allele associated with B-lymphocytes abnormal accumulation and increased risk of multiple myeloma^16^. Distribution of the protective allele in the overall Italian population thus seems to have been favoured by natural selection having acted on it or by hitchhiking effect related to unknown advantageous variants. Interestingly, *TNKS* plays a role in the pathway of downstream signalling that originates from B cell receptors after their interaction with antigens and that mediates peptides internalization and presentation to T helper cells, thus ensuring proper antibodies production^17^.

Ten genomic intervals encompassing ten different genes and one regulatory region were retained as the most plausible targets of positive selection in S_ITA (Supplementary Table S5) and were shared especially with people from C_ITA. In addition to the above-mentioned signals surrounding the *DLEU1* lincRNA and *TNKS* enancher sequence, the most relevant S_ITA signature involved part of the *IL23R* locus, extending on a considerably larger chromosomal interval with respect to what observed in C_ITA. All putatively selected *IL23R* derived alleles are associated to increased risk of developing inflammatory phenotypes, such as inflammatory bowel and Crohn's diseases^18-20^. These chronic disorders are caused by the interplay of several environmental, immune and bacterial factors acting on genetically susceptible individuals and resulting in aggressive immune responses to possible microbial antigens, which instead damage the own cells of the gastrointestinal tract^21^. We can thus hypothesize that part of such a genetic susceptibility might be related to alleles previously targeted by natural selection according to their capability to confer aggressive responses to pathogens, but that became detrimental after the recent cultural shifts responsible for introduction of completely new immune-stimulatory epitopes in the diets of western societies^22^. In fact, *IL23R* encodes for a subunit of the receptor of pro-inflammatory cytokine interleukin-23 that activates effector T cells. Therefore, it perpetrates organ-specific inflammation and plays a key role in modulating responses to pathogens, especially in the case of mycobacterial infections^23^. According to this evidence, as well as considering the impact of identified candidate SNPs on *IL23R* functionality and on the strength of the related inflammatory cascade, we can speculate that specific infectious diseases, among which tuberculosis caused by *Mycobacterium tuberculosis* and leprosy caused by *Mycobacterium leprae*, could have represented selective pressures able to considerably shape Italian variation at this gene. This hypothesis is compatible with the long coexistence between human populations settled along the Italian peninsula and such mycobacteria, as proved by the Italian paleoanthropological record that provided the earliest evidence of tuberculosis in the human species dated to around 5,800 years ago, as well as proofs of individuals affected by leprosy since approximately 2,400 years ago^24^.

Eight different genomic windows including seven genes and one regulatory sequence were then identified as candidate chromosomal intervals to have undergone positive selection in SARD, with three of them being not shared with population groups from peninsular Italy (Supplementary Table S5). The most relevant SARD specific signature was observed at an intergenic region located between the *LOC100049717* and *TYRP1* coding sequences. It involved a variant (rs4008031) listed among the top non-HLA SNPs showing significant nominal p-values for association to celiac disease^25^, thus suggesting its potential role as modulator of T lymphocytes responses to antigens. Moreover, *TYRP1* encodes a melanosomal enzyme involved in mammalian skin and/or hair pigmentation so that some of its variants were proved to determine blond hair in Solomon Islanders and human oculocutaneous albinism type 3^26^. However, despite the potential link between such known phenotypes and the observed adaptive signature, low LD was confirmed in TSI between *TYRP1* functional SNPs and the identified loci putatively targeted by selection. A second signal restricted to SARD emerged at a genomic window covering part of the *CR1* gene. This locus encodes for a protein that prevents immune complex deposition in the vessel wall by clearing complement-tagged inflammatory particles from the circulation and the putative selected allele at one of its identified outlier SNPs (rs12034383) is a missense variant able to influence erythrocytes sedimentation rate^27^. Since the clearance of immune complexes is involved in the pathogenesis of malaria, such a kind of SNPs was supposed to affect inter-individual variation in rosetting, a process resulting from adhesion of *Plasmodium falciparum* erythrocyte membrane protein 1 located on the surface of infected cells to uninfected erythrocytes, thus contributing to the severity of malaria^28^. In fact, variation at *CR1* was already proposed to modulate the malarial phenotype in populations from Papua New Guinea and an African-specific allele (rs17047661) was proved to be subjected to positive selection plausibly due to its capability to modify host disease susceptibility^29^. However, this peculiar allele is nearly absent in Italy (0.5%) and in extremely low LD with the putative *CR1* selected alleles identified in the Italian population. Interestingly, also Parolo et al.^30^ detected a signature of positive selection at such gene in a generic sample of Italians. However, they applied less stringent iHS thresholds with respect to those used in the present study and did not report the observed candidate SNPs. Moreover, they did not identify in detail which Italian population groups have been targeted by selection. Conversely, according to our conservative approach a significant footprint of selection emerged only in SARD and on *CR1* ancestral alleles that show considerably larger frequencies in such a group than in peninsular Italians (on average, 73% vs 26%). It can be thus hypothesized that although the selective pressure exerted by malaria acted also on some populations settled along the peninsula, it played a more outstanding role in shaping variation of the Sardinian people, as suggested also by previous studies^2^. A third SARD specific signature was observed at a genomic window including two variants located upstream of the *MS4A2* gene encoding for subunit beta of the immunoglobulin epsilon receptor that is mainly expressed on the surface of mast cells and basophils and that is involved in allergic responses. Derived alleles at such SNPs (rs574700 and rs1441585) lay within a haplotype associated to increased risk of asthma and are in strong LD (r^2^ = 0.92) in TSI with rs569108, the variant putatively responsible for such association signal^31^. These alleles are nearly fixed in most European and peninsular Italian populations, while their frequency decreases to 87% in SARD. Positive selection seems to have acted in this human group on the complementary ancestral alleles of such loci, plausibly in response to the presence of geographically restricted environmental toxins or allergens, making Sardinians less susceptible to their harmful effects. Since the same functional pathway targeted by the antimalarial drug *artesunate* largely contributes to the production of pro-inflammatory and oxidative metabolites involved in the development of asthma^32^, we can speculate that a malaria-related selective pressure could be responsible also for *MS4A2* Sardinian adaptive evolution in addition to *CR1* one*.*

Furthermore, a number of less outstanding adaptive events seem to be shared, partially or completely, by the examined population groups. For instance, a signature observed in all Italian populations except those from N_ITA targeted a genomic region including an intron of the *ELMO1* gene and involved two genic SNPs in S_ITA, as well as a single genic SNP and an intergenic variant in C_ITA and SARD. Among such SNPs, rs2041801 is associated to diabetic nephropathy, one of the major complications of both type 1 and type 2 diabetes^33^. Although no functional data are available for this SNP, overexpression of *ELMO1* is proved to increase transcription of growth factor-β, collagen type 1 and fibronectin, thus modulating inflammatory responses and improving healing of inflammatory lesions^33^. This pattern could have represented an advantageous trait in certain past pathogens’ landscapes, suggesting that rs2041801 and/or variants in high LD with it have been subjected to positive selection due to their impact on *ELMO1* expression. However, when coupled with the high glucose levels typical of modern diabetic phenotypes such a condition accelerates progression of chronic glomerular injury through dysregulation of extracellular matrix metabolism, finally leading to impaired renal functionality. Three additional footprints of positive selection were instead shared among all peninsular populations, but not with SARD. A first signal was detected at a window including one downstream SNP and two missense variants on *CNPPD1*, a gene involved in modulation of cyclin-dependent protein serine/threonine kinase activity that contributes to processes of cell cycle regulation and neuronal differentiation. A chromosomal interval encompassing only the two non-synonymous SNPs scored in the top 0.1% of windows enriched for outlier loci also in C_ITA and S_ITA. However, these variants are not known to have a specific functional impact on the CNPPD1 protein, nor to be in strong LD with SNPs showing established phenotypic effects. A further footprint of selection highlighted a genomic region encompassing two missense SNPs at *TLR10*, the sole gene exerting an anti-inflammatory role among those belonging to the Toll-like receptor family devoted to sense microbes and to modulate inflammatory reaction against them^34^. Such a gene was already proposed as a target of positive selection in the Italian population, but putative adaptive SNPs were not pinpointed^30^. Conversely, we identified candidate selected alleles at rs11096955 and rs11096957 variants that have no functional impact on the TLR10 protein, but that in TSI are in moderate LD (r^2^ = 0.60) with the *TLR1* rs5743618 allele proved to be protective against leprosy^35^. Moreover, these SNPs lay on the same haplotypic background at the *TLR10*-*TLR1*-*TLR6* gene cluster that has been shown to have undergone adaptive evolution in populations of European ancestry, with rs5743618 representing the functional SNP more plausibly targeted by selection^36^. A third shared signature identical in N_ITA and C_ITA, but involving two additional regulatory SNPs in S_ITA, was observed at a window including one missense substitution at *BAG6* and a regulatory SNP on an enancher sequence of the *LY6G6C* gene. These loci cluster on the major histocompatibility complex class III region. The former plausibly plays a role in immuno-proteasomes to degrade antigens and to enable peptides presentation to immune effectors, while the latter encodes for a lymphocyte antigen complex mainly expressed in the skin and implicated in signal transduction from the cell surface. In particular, *BAG6* rs1052486 was found to be in complete LD in TSI with rs1052486, which represents a risk allele for the common autoimmune inflammatory disease systemic lupus erythematosus, while *LY6G6C* rs805294 was directly associated to such a pathological condition^37,38^. This could be interpreted as a further clue corroborating the hypothesis of maladaptation assuming that some loci relevant for contrasting pathogens have been positively selected in the past, but may contribute to increased susceptibility to autoimmune disorders in present-day populations, as suggested above for *IL23R*.

Finally, three plausible adaptive events seem to have occurred in all Italian population groups, SARD included. A first one involved two intronic SNPs on the *ESR1* gene encoding for an estrogen-activated transcription factor mainly expressed in epithelial cells and essential for reproductive functions. However, no data about functional impact of these candidate SNPs are available in literature. Moreover, they did not turn out to be in high LD in TSI with variants known to be associated to reduced risk of endometrial and breast cancers and plausibly exerting a regulatory action on *ESR1* expression^39^. A second region scoring in the top 0.1% of windows enriched for outlier SNPs encompassed one missense SNP on the *PTGR1* gene in all Italian population groups, further extending to an additional intronic variant in S_ITA. Again, no functional evidence is available for such candidate SNPs, as well as for variants in high LD with them. However, the PTGR1 protein is known to catalyse metabolic inactivation of leukotriene B4, a pro-inflammatory chemoattractant produced by leukocytes and responsible for both neutrophils recruitment on endothelia and induction of reactive oxygen species formation useful as antimicrobial response. In particular, according to recent evidence of *PTGR1* overexpression being able to restrict inflammation and confer increased resistance to tuberculosis^40^ we can again speculate that *Mycobacterium tuberculosis* might have represented a remarkable selective pressure for past Italian populations, in line with some of the above-described results. A third signal was finally detected at a genomic window including the *CYBRD1* gene, which encodes an iron-regulated protein mainly expressed in the brush border membrane of duodenal enterocytes and that exerts ferric reductase activity, being implicated in absorption of dietary iron and regulation of iron homeostatsis^41^. However, also in such a case no *in silico* predictions and/or experimental evidence describing the potential effects of the identified candidate variants were available in literature.

Due to the availability of an independent dataset including Italian samples representative of population clusters identified in the present study, but typed for 2.5 M markers^4^, we replicated iHS analysis on 200 kb chromosomal intervals centred on our top candidate targets of selection. However, only signals detected at non-genic sequences located near *ELMO1*, as well as between the *LOC100049717* and *TYRP1* genes, were successfully replicated in C_ITA and SARD, respectively. Overlap of significant results between the two datasets thus appears to be scarce, plausibly because it is restricted to intergenic SNPs, a class of loci that drove a small proportion of the independent selection signals pointed out by our analysis (6%) with respect to what observed for non-synonymous (31%) or regulatory (63%) SNPs. In the present study, we indeed found that genic variants were enriched in selection signals when compared to non-genic ones (χ^2^ test, p = 3.05x10^-5^) and that an excess of outliers in non-synonymous versus synonymous (i.e. non-genic and genic silent substitutions) SNPs was also observable (χ^2^ test, p = 6.11x10^-6^). These findings likely result from the peculiar rationale underlying the design of the CoreExomeChip array, which is devoted to assay mainly functional variation by implementing, in addition to the Illumina Core selection of genome-wide tag SNPs, also 250,000 exomic SNPs and 20,000 markers with known functional or regulatory impact on loci involved in disease phenotypes. Instead, genomic regions harbouring such variants were scarcely represented in the HumanOmni2.5-BeadChip array, thus preventing us to obtain reliable validation of our top candidate signatures of positive selection.

**Additional Loci Influencing Differential Disease Susceptibility Along the Italian Peninsula**

No variants showed significant ∆DAF when C_ITA was compared with both northern and southern clusters. Conversely, when the analysis was extended to non-peninsular Italians, a number of relevant loci were pointed out providing further evidence for the peculiar genetic background of Sardinians with respect to that of the overall Italian population. In particular, 68 loci with ∆DAF ranging from 0.10 to 0.48 (p < 3.12x10^-8^) and with average Fst value of 0.25 were observed when contrasting the SARD and C_ITA clusters, 34 SNPs (∆DAF = 0.14-0.50, p < 3.31x10^-8^, average Fst = 0.26) were observed when the N_ITA group was considered and 14 (∆DAF = 0.15-0.44, p < 3.25x10^-8^, average Fst = 0.26) when the comparison involved S_ITA.

**Supplementary Methods**

**Data Curation**

Stringent per-individual QC procedures were performed by removing samples showing more than 1% missing genotypes (n = 4) and discordant genetic/ascertained sex (n = 3), as revealed by comparison of each individual’s mean homozygosity rate across X-chromosome markers with the expected rate (i.e. rate less than 0.2 for females and above 0.8 for males). As a measure of the quality of typed DNA samples, autosomal heterozygosity rate per individual was also assessed and subjects with excessive or reduced proportion of heterozygote genotypes (i.e. with values exceeding ± 3 standard deviations from the mean) were removed (n = 5).

An Italian LD-pruned dataset containing 135,905 variants showing extremely reduced LD with each other was created by removing regions of extended LD on 11 chromosomes^42^ and by pruning one marker from each remaining pair showing r^2^ values higher than 0.2 according to a sliding windows approach with window size of 50 variants and sliding 5 loci sequentially.

The extended dataset included samples typed with different genotyping arrays so that after merging and QC procedures a subset of 110,162 variants overlapping in the present and the considered studies was retained. To avoid the achievement of biased results from multivariate and admixture analyses, also this dataset was subsequently pruned for LD according to the above-mentioned procedure. The final extended LD-pruned dataset included 55,029 variants.

**Population Structure Analyses**

A first explorative PCA pointed out 10 Italian individuals as outliers with respect to samples collected in their province of origin according to eigenvectors for one of the most informative PCs exceeding ± 3 standard deviations from the mean calculated for that sampling location. PCA was thus repeated on both the Italian and extended datasets by including only 737 non-outlier Italian subjects.

Procrustes analysis was used to performed single value decomposition between different axes (i.e. PCA eigenvectors and longitude/latitude coordinates) to optimize transformation of the first to the second set of coordinates.

DAPC was repeated with different randomized groups for different amounts of retained PCs to identify their optimal number as that optimizing the mean α-score. Linear discriminant analysis was finally performed on the optimized number of retained PCs to minimize within-groups variance and to maximize between-groups one, for depicting genetic relationships among population clusters suggested by PCA and Procrustes analyses. Given the low number of tested clusters, all discriminant functions were retained to perform the analysis.

**Admixture Analyses**

TreeMix was first used to construct a tree without migration to evaluate overall topology and extent of drift in terms of allele frequency shift from an ancestral population. Inspection of the matrix of residuals from the fit of the model to the data was then used to point out population pairs that could be better described by including migration into the model. Re-organization of the tree with inclusion of increasing migration edges was finally performed to account for admixture events suggested by analysis of residuals.

**Detection of Genomic Signatures of Natural Selection**

***Integrated Haplotype Score Analysis***

Only 200 kb windows with more than 10 SNPs and binned according to their number of loci with a 10 SNPs increase per bin were considered. Rank p-values were then computed for each window as the fraction of them showing greater proportion of outlier SNPs in that bin according to both F_st_ and |iHS| values (i.e. by dividing the number of windows with a greater proportion of extreme SNPs with respect to the tested window by the number of windows in the considered bin). Windows scoring in the bottom 0.1% of obtained p-values distributions were finally retained for each population cluster as the most plausible candidate regions that have undergone positive selection.

***Derived Allele Frequency Comparison***

Combination of F_st_ and ∆DAF analyses is expected to be particularly suited for the identification of soft sweeps occurring on standing variation and assessment of ∆DAF has been recently proved to be highly effective in detecting both complete and partial selective sweeps, even outperforming some haplotype-based tests (e.g. XP-EHH) in the recognition of the latter^43^.

**Supplementary References**

1. Contu, D. *et al.* Y-chromosome based evidence for pre-neolithic origin of the genetically homogeneous but diverse Sardinian population: inference for association scans. *PLoS ONE* **3,** e1430 (2008).
2. Piras, I. S. *et al.* Genome-wide scan with nearly 700,000 SNPs in two Sardinian sub-populations suggests some regions as candidate targets for positive selection. *Eur. J. Hum. Genet.* **20,** 1155–1161 (2012).
3. Di Gaetano, C. *et al.* Sardinians genetic background explained by runs of homozygosity and genomic regions under positive selection. *PLoS ONE* **9,** e91237 (2014).
4. Fiorito, G. *et al.* The Italian genome reflects the history of Europe and the Mediterranean basin. *Eur. J. Hum. Genet.* (2015) doi:10.1038/ejhg.2015.233.
5. Sikora, M. *et al.* Population genomic analysis of ancient and modern genomes yields new insights into the genetic ancestry of the Tyrolean Iceman and the genetic structure of Europe. *PLoS Genet.* **10,** e1004353 (2014).
6. Lao, O. *et al.* Correlation between Genetic and Geographic Structure in Europe. *Curr. Biol.* **18,** 1241–1248 (2008).
7. Moran, V.A., Perera, R.J. & Khalil, A.M. Emerging functional and mechanistic paradigms of mammalian long non-coding RNAs. *Nucleic Acids Res.* **40,** 6391–6400 (2012).
8. Heward, J.A. & Lindsay, M.A. Long non-coding RNAs in the regulation of the immune response. *Trends Immunol.* **35,** 408–419 (2014).
9. Hu, Y. *et al.* RP5-833A20.1/miR-382-5p/NFIA-dependent signal transduction pathway contributes to the regulation of cholesterol homeostasis and inflammatory reaction. *Arterioscler Thromb Vasc Biol.* **35,** 87–101 (2015).
10. Ross, A., Beales, P.L. & Hill J. The Clinical, Molecular, and Functional Genetics of Bardet–Biedl Syndrome, in Genetics of Obesity Syndromes (eds. Beales, P.R., Farooqi, I.S., O'Rahilly, S.) (Oxford University Press, 2008).
11. Sabatti, C. *et al.* Genome-wide association analysis of metabolic traits in a birth cohort from a founder population. *Nat Genet*. **41,** 35–46 (2009).
12. St-Onge, M.P., Zhang, S., Darnell, B., & Allison, D.B. Baseline serum C-reactive protein is associated with lipid responses to low-fat and high-polyunsaturated fat diets. *J Nutr*. **139,** 680–683 (2009).
13. Sabater-Lleal, M. *et al.* Multiethnic meta-analysis of genome-wide association studies in >100000 subjects identifies 23 fibrinogen-associated loci but no strong evidence of a causal association between circulating fibrinogen and cardiovascular disease. *Circulation*. **128,** 1310–1324 (2013).
14. Wolf, S. *et al.* B-cell neoplasia associated gene with multiple splicing (BCMS): the candidate B-CLL gene on 13q14 comprises more than 560 kb covering all critical regions. *Hum Mol Genet*. **10,** 1275–1285 (2001).
15. Weedon, M.N. *et al.* Genome-wide association analysis identifies 20 loci that influence adult height. *Nat Genet.* **40,** 575–583 (2008).
16. Weinhold, N. *et al.* The CCND1 c.870G>A polymorphism is a risk factor for t(11;14)(q13;q32) multiple myeloma. *Nat Genet.* **45,** 522-525 (2013).
17. Seda, V. & Mraz, M. B-cell receptor signalling and its crosstalk with other pathways in normal and malignant cells. *Eur J Haematol.* **94,** 193–205 (2015).
18. Magyari, L., Kovesdi, E., Sarlos, P., Javorhazy, A., Sumegi, K. & Melegh, B. Interleukin and interleukin receptor gene polymorphisms in inflammatory bowel diseases susceptibility. *World J Gastroenterol.* **20,** 3208–3222 (2014).
19. Raelson, J.V. *et al.* Genome-wide association study for Crohn's disease in the Quebec Founder Population identifies multiple validated disease loci. *Proc Natl Acad Sci USA*. **104,** 14747–14752 (2007).
20. Wellcome Trust Case Control Consortium. Genome-wide association study of 14,000 cases of seven common diseases and 3,000 shared controls. *Nature*. **447,** 661–678 (2007).
21. Baumgart, D.C. & Sandborn, W.J. Crohn's disease. *Lancet* **380,** 1590–1605 (2012).
22. Sazzini, M. *et al.* Ancient pathogen-driven adaptation triggers increased susceptibility to non-celiac wheat sensitivity in present-day European populations. *Genes Nutr*. **11,** 15 (2016).
23. Frank, A. *et al.* Human IL-23-producing type 1 macrophages promote but IL-10-producing type 2 macrophages subvert immunity to (myco)bacteria. Proc Natl Acad Sci USA **101,** 4560–4565 (2004).
24. Stone, A.C., Wilbur, A.K., Buikstra, J.E. & Roberts, C.A. Tuberculosis and leprosy in perspective. *Am J Phys Anthropol.* **49,** 66–94 (2009).
25. Hunt, K. C. *et al.* Novel celiac disease genetic determinants related to the immune response. *Nat Genet.* **40,** 395–402 (2008).
26. Kenny, E.E. *et al.* Melanesian blond hair is caused by an amino acid change in TYRP1. *Science*. **336**, 554 (2012).
27. Kullo, I.J. *et al.* Complement Receptor 1 Gene Variants Are Associated with Erythrocyte Sedimentation Rate. *Am J Hum Genet.* **89,** 131–138 (2011).
28. Thathy, V., Moulds, J.M., Guyah, B., Otieno W. & Stoute J.A. Complement receptor 1 polymorphisms associated with resistance to severe malaria in Kenya. *Malar J.* **4,** 54 (2005).
29. Barreiro, L.B., Laval, G., Quach, H., Patin, E. & Quintana-Murci L. Natural selection has driven population differentiation in modern humans. *Nat Genet*. **40,** 340–345 (2008).
30. Parolo, S. *et al.* Characterization of the biological processes shaping the genetic structure of the Italian population. *BMC Genet*. **16,** 132 (2015).
31. Joubert, B.R. *et al.* Evaluation of genetic susceptibility to childhood allergy and asthma in an African American urban population. *BMC Med Genet.* **12,** 25 (2011).
32. Ho, W. E., Cheng, C., Peh, H. Y., Xu, F., Tannenbaum, S. R., Ong, C. N. & Wong, W. S. Anti-malarial drug artesunate ameliorates oxidative lung damage in experimental allergic asthma. *Free Radic Biol Med.* **53,** 498–507 (2012).
33. Pezzolesi, M. G. *et al.* Confirmation of genetic associations at ELMO1 in the GoKinD collection supports its role as a susceptibility gene in diabetic nephropathy. *Diabetes* **58,** 2698–2702 (2009).
34. Oosting, M. *et al.* Human TLR10 is an anti-inflammatory pattern-recognition receptor. *Proc Natl Acad Sci U S A.* **111,** E4478–4484 (2014).
35. Hart, B.E. & Tapping, R.I. Differential trafficking of TLR1 I602S underlies host protection against pathogenic mycobacteria. *J Immunol*. **189,** 5347–5355 (2012).
36. Barreiro, L. B. *et al.* Evolutionary dynamics of human Toll-like receptors and their different contributions to host defense. *PLoS Genet*. **5,** e1000562 (2009).
37. International Consortium for Systemic Lupus Erythematosus Genetics (SLEGEN) *et al.* Genome-wide association scan in women with systemic lupus erythematosus identifies susceptibility variants in ITGAM, PXK, KIAA1542 and other loci. *Nat Genet.* **40,** 204–210 (2008).
38. Fernando, M. M. *et al.* Identification of two independent risk factors for lupus within the MHC in United Kingdom families. *PLoS Genet*. **3,** e192 (2007).
39. Einarsdóttir, K. *et al.* Common genetic variability in ESR1 and EGF in relation to endometrial cancer risk and survival. *Br J Cancer.* **100,** 1358–1364 (2009).
40. Tobin, D. M., Roca, F. J., Ray, J. P., Ko, D. C. & Ramakrishnan, L. An Enzyme That Inactivates the Inflammatory Mediator Leukotriene B4 Restricts Mycobacterial Infection. *PLoS One* **8,** e67828 (2013).
41. McKie, A.T. *et al.* An iron-regulated ferric reductase associated with the absorption of dietary iron. *Science* **291,** 1755–1759 (2001).
42. Price *et al.* Long-range LD can confound genome scans in admixed populations. *Am. J. Hum. Genet*. **83,** 132 (2008).
43. Colonna, V. *et al.* Human genomic regions with exceptionally high levels of population differentiation identified from 911 whole-genome sequences. *Genome Biol.* **15,** R88 (2014).
